# Proteolysis of mature HIV-1 p6 Gag protein by the insulin-degrading enzyme (IDE) regulates virus replication in an Env-dependent manner

**DOI:** 10.1371/journal.pone.0174254

**Published:** 2017-04-07

**Authors:** Friedrich Hahn, Adrian Schmalen, Christian Setz, Melanie Friedrich, Stefan Schlößer, Julia Kölle, Robert Spranger, Pia Rauch, Kirsten Fraedrich, Tatjana Reif, Julia Karius-Fischer, Ashok Balasubramanyam, Petra Henklein, Torgils Fossen, Ulrich Schubert

**Affiliations:** 1 Institute of Virology, Friedrich-Alexander-University Erlangen-Nürnberg, Erlangen, Germany; 2 Translational Metabolism Unit, Diabetes Research Center, Division of Diabetes, Endocrinology and Metabolism, Baylor College of Medicine, Houston, Texas, United States of America; 3 Institute of Biochemistry, Charité Universitätsmedizin-Berlin, Berlin, Germany; 4 Department of Chemistry and Centre for Pharmacy, University of Bergen, Bergen, Norway; "INSERM", FRANCE

## Abstract

There is a significantly higher risk for type II diabetes in HIV-1 carriers, albeit the molecular mechanism for this HIV-related pathology remains enigmatic. The 52 amino acid HIV-1 p6 Gag protein is synthesized as the C-terminal part of the Gag polyprotein Pr55. In this context, p6 promotes virus release by its two late (L-) domains, and facilitates the incorporation of the viral accessory protein Vpr. However, the function of p6 in its mature form, after proteolytic release from Gag, has not been investigated yet. We found that the mature p6 represents the first known viral substrate of the ubiquitously expressed cytosolic metalloendopeptidase insulin-degrading enzyme (IDE). IDE is sufficient and required for degradation of p6, and p6 is approximately 100-fold more efficiently degraded by IDE than its eponymous substrate insulin. This observation appears to be specific for HIV-1, as p6 proteins from HIV-2 and simian immunodeficiency virus, as well as the 51 amino acid p9 from equine infectious anaemia virus were insensitive to IDE degradation. The amount of virus-associated p6, as well as the efficiency of release and maturation of progeny viruses does not depend on the presence of IDE in the host cells, as it was shown by CRISPR/Cas9 edited IDE KO cells. However, HIV-1 mutants harboring IDE-insensitive p6 variants exhibit reduced virus replication capacity, a phenomenon that seems to depend on the presence of an X4-tropic Env. Furthermore, competing for IDE by exogenous insulin or inhibiting IDE by the highly specific inhibitor 6bK, also reduced virus replication. This effect could be specifically attributed to IDE since replication of HIV-1 variants coding for an IDE-insensitive p6 were inert towards IDE-inhibition. Our cumulative data support a model in which removal of p6 during viral entry is important for virus replication, at least in the case of X4 tropic HIV-1.

## Introduction

The 52 aa HIV-1 p6 protein is one of the smallest known lentiviral proteins. It is synthesized as the C-terminal part of the Gag polyprotein Pr55 and is released by viral protease (PR) during virus morphogenesis. It is abundantly present in progeny virions and after virus disintegration should be present in less characterized intra- and extracellular compartments, where its abundance and function have not been defined yet. The Gag polyprotein Pr55^Gag^ is necessary and sufficient for virus-like particle (VLP) formation [1,2]. At the time of virus release, Gag is processed by PR into the structural components matrix (MA), capsid (CA), nucleocapsid (NC), and p6, as well as two spacer peptides SP1 and SP2.

Many functions have been ascribed to the C-terminal p6 region of Gag during late steps of virus replication, involving assembly, release, and maturation of progeny virions. Two late (L-) domains, the P(T/S)AP and YP(X)_n_L motifs, mediate the abscission of budding virions from the plasma membrane and serve as docking sites for components of the cellular endosomal sorting complex required for transport (ESCRT). The ESCRT machinery normally mediates topologically equivalent membrane fission events during membrane protein trafficking and cytokinesis [3,4,5,6]. Furthermore, the highly conserved LXXLF motif within the C-terminus of p6 governs the incorporation of viral protein R (Vpr) into budding virions [7,8].

Various post-translational modifications have been ascribed to p6, including SUMOylation at lysine 27 and mono-ubiquitination at lysines 27 and 33 [9,10,11]. Additionally, p6 is the predominant phosphoprotein of HIV-1 particles [12]. The biological relevance of these modifications of p6 remains to be elucidated [10,13,14].

Beyond its functions in the context of Pr55, almost nothing is known about the role of the mature p6 protein. It is present in amounts equimolar to CA in virions, and thus should account for 2000–5000 molecules [15,16] per released virion, the destiny of which is either to infect a new host cell or to disintegrate in the extracellular space. Up to 10^10^ HIV-1 particles are produced and disintegrated per day accounting for up to 50 pg of CA antigen per ml plasma in high viraemic HIV-1 infected persons. However, the fate of free p6, either as part of a mature virion infecting a new host cell or stemming from decayed virions in the extracellular space has not been investigated yet. Although free p6 can be found in mature virions, it is hardly detectable in infected cells or in cell-free spaces, raising the question about the fate of p6 outside an HIV-1 particle.

Here, we demonstrate that HIV-1 p6 in its mature form is specifically cleaved by a ubiquitously expressed cytosolic metalloendopeptidase, the insulin-degrading enzyme (IDE) or insulysin, with a ~100-fold higher efficiency compared to its physiologic substrate insulin. Furthermore, an IDE-specific inhibitor, 6bK, as well as exogenous insulin, interfere with X4-tropic HIV-1 replication in activated PBMCs, most probably by competing with p6 for degradation by IDE. In consistency, an IDE-insensitive p6 mutant of HIV-1 exhibits impaired replication capacity but is insensitive to the treatment with insulin or 6bK. Conversely, neither virus release and maturation, nor the amounts of particle-associated Vpr and p6 itself were altered in IDE knock out cells. Thus, our cumulative data support a model in which IDE is responsible for the rapid degradation of p6 entering the cell as part of the incoming virion, a process that appears to be crucial to achieve optimal X4-tropic virus replication.

## Results

### The majority of mature p6 is present in virus particles

**Fig 1:**
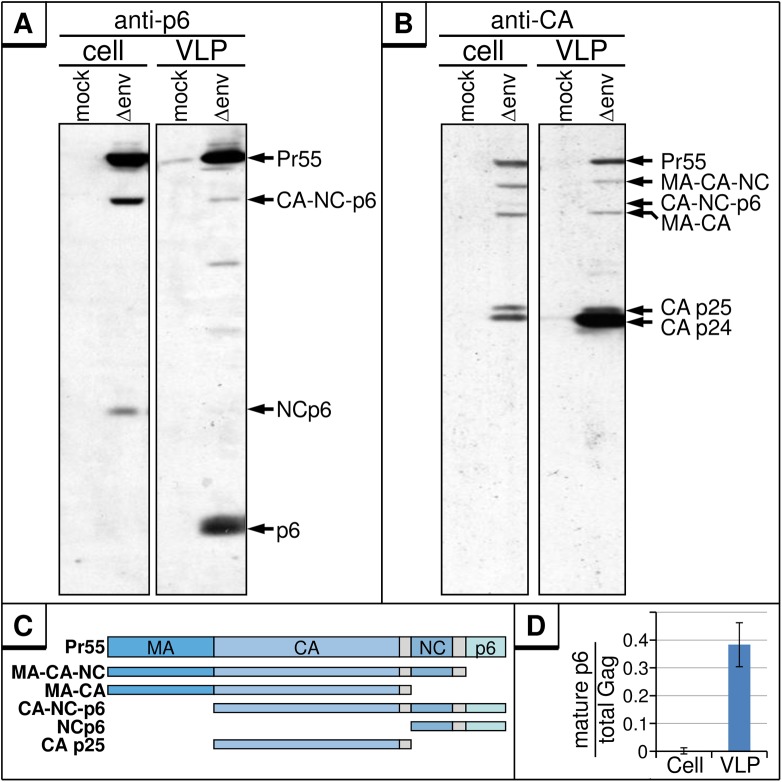
Mature p6 is detectable only in released virus particles. HEK293T cells were transfected with the *env*-deleted HIV-1 expression plasmid pNL*env*1 (Δenv). Cell lysates and VLP fractions were analyzed by Western blotting using a p6-reactive (**A**) or a CA-reactive (**B**) antiserum. (**C**) Schematic depiction of Gag processing products observable in A and B. (**D**) Quantitative analysis of mature p6 versus total amount of p6-containing Gag proteins for cell and VLP fractions. Values represent the arithmetic mean of 3 independently performed experiments ± SD.

Hitherto, all functions of p6 have been attributed to those of its parental precursor protein, the Gag polyprotein Pr55, and almost nothing is known about the role of mature p6 in the HIV-1 life cycle. Hence, we first analyzed the relative content of free p6 in cell and virus fractions. To obtain high expression levels of Gag, HEK293T cells were transiently transfected with the subgenomic HIV-1 expression plasmid pNL*env*1 (Δenv) [17] which directs efficient expression of all HIV-1 proteins except the Env glycoproteins. Western blot analyses of cell and VLP fractions revealed significant amounts of p6 in VLP fractions (Fig 1A, right panel) as well as the other p6-containing Gag processing intermediates (Fig 1C). Conversely, mature p6 was absent intracellularly, despite of the fact that a similar pattern of Gag proteins was detectable with anti-CA antibody (Fig 1B, left panel). As occasionally observed by us and others, only traces of p6, ~100-fold less than in virus fractions, were detected intracellularly in repeated experiments (Fig 1A, left panel, 1D) [18].

The virtual absence of intracellular p6 might be due to insufficient processing of the NCp6 intermediate before detachment of the virus, consistent with reports demonstrating that NCp6 is cleaved in a late step of virus budding which requires RNA-binding of the NC part within NCp6 [19]. Alternatively, the loss of p6 could result from a selective instability of p6 within cells.

**Fig 2:**
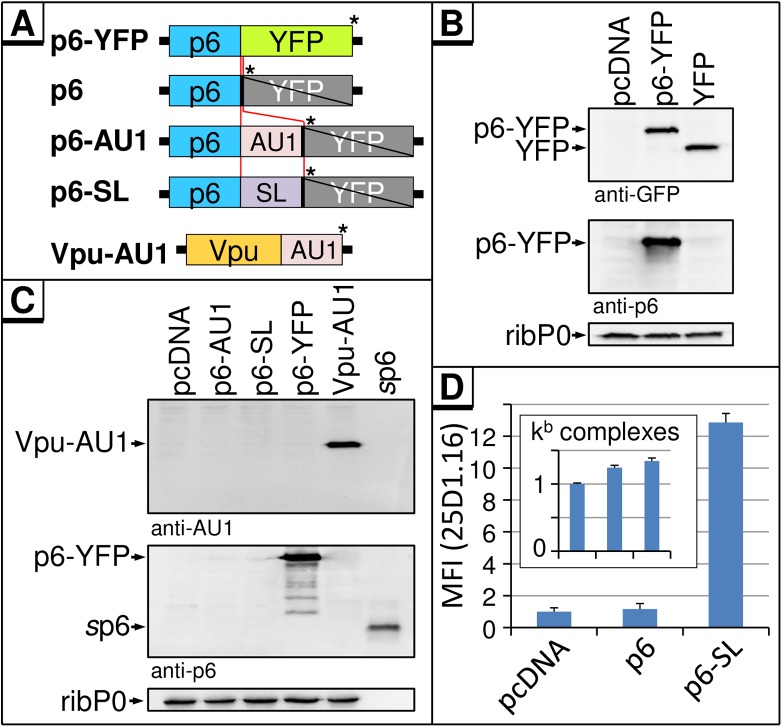
p6 is expressed as a YFP-fusion, but is highly turned over when expressed as an autonomous protein. **(A)** Schematic depiction of the CMV-driven expression plasmids. For ectopic expression of p6, a codon-optimized *p6* gene was fused to YFP and cloned into the vector pcDNA (p6-YFP). By insertion of a stop codon (*) after the *p6* ORF, translation of YFP-fusion part was abrogated. Additional vectors were established with an AU1 tag or a SIINFEKL (SL)-epitope at the C-terminus of the *p6* ORF. For control, the Vpu-AU1 construct encodes a Vpu protein harboring an AU1 tag. **(B)** HeLa cells were transfected with plasmids indicated and expression of p6-YFP fusion was detected with antibodies specific for GFP or p6. Staining for the ribosomal P-antigen (ribP0) served as loading control. **(C)** Cytosolic extracts from HeLa cells transfected with indicated constructs were analyzed by Western blotting using antibodies specific for AU1 or p6. 20 ng of *s*p6 served as control for the anti-p6 staining. **(D)** HeLa-K^b^ cells were transfected with p6-expression constructs, coding for p6 or p6-SL and as an empty vector control pcDNA. H2-K^b^-SL complexes presented on the cell surface were quantified by flow cytometry using the mAb 25D1.16-APC. Inset: the total amount of H2-K^b^ complexes was determined by the mAb B8-24-3. Quantification of five independent experiments; Bars represent mean values ± SD.

To challenge this hypothesis, we tried to express p6 in its mature form from a codon-optimized transgene. This has never been reported before, and attempts to express p6 in HEK293T cells failed [10]. For ectopic expression of p6, plasmids directing the expression of a p6-YFP fusion protein (p6-YFP) or p6 tagged with either an AU1 tag (p6-AU1) or the ovalbumin-derived MHC-I model epitope SIINFEKL (SL) [20] (p6-SL) have been established (Fig 2A). p6-YFP directs the expression of a fusion protein which reacted with both anti-p6 and anti-GFP antibodies (Fig 2B). However, no expression of free p6 was detectable by Western blot when p6-AU1 or p6-SL were transfected, although the respective controls Vpu-AU1 [21], p6-YFP, and p6 synthesized by solid phase peptide synthesis (*s*p6) [18] were readily detected (Fig 2C). To investigate whether p6 is expressed at all, the MHC-I antigen presentation of p6 derived epitopes was determined, a very sensitive method that has been established to demonstrate the expression of highly turned-over Gag proteins [22,23,24,25]. As there are no antibodies available that recognize p6-derived epitopes bound to human MHC-I molecules, SL was introduced *in frame* at the C-terminus of p6. HeLa cells stably expressing the murine MHC-I allotype H2-K^b^ (HeLa-K^b^) [26] were transfected with expression plasmids coding for *wt* p6, p6-SL or the empty vector (pcDNA). Flow cytometry using the mAb 25D1.16, which specifically recognizes H2-K^b^-bound SL, revealed that only cells expressing p6-SL displayed a readily detectable 25D1.16 staining indicative for the presence of H2-K^b^-SL complexes at the cell surface (Fig 2D), while the total amounts of H2-K^b^ complexes were similar [27] (Fig 2D inset). These results indicate that the complete amino acid sequence of p6 was expressed, yet the protein itself was not detectable by Western blot (Fig 2C).

### A cytosolic enzymatic activity specifically degrades p6

**Fig 3:**
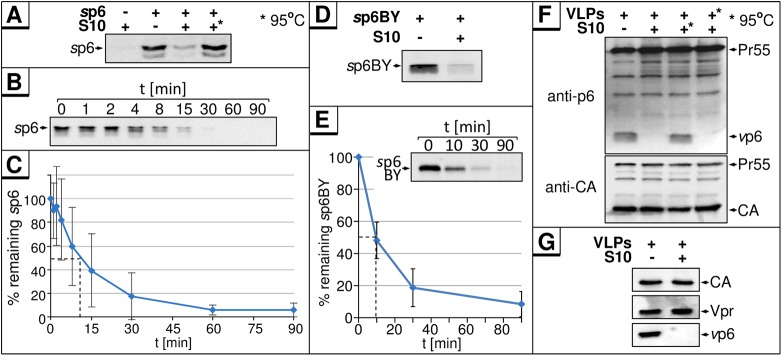
Cytoplasmic S10 from HeLa cells contains an enzymatic activity that degrades *s*p6 and *v*p6. **(A)** 100 ng *s*p6 were incubated with 5 μg S10 extract from HeLa cells for 30 min at 37°C. In one reaction, S10 extract was heat-inactivated (95°C, 5 min) prior to incubation (*). **(B)** 100 ng *s*p6 were incubated with 5 μg S10 extract for the times indicated at 37°C. **(C)** Amounts of p6 were quantified for four independently performed experiments. Values represent the arithmetic mean ± SD. **(D)** 10 ng *s*p6BY were incubated with 5 μg S10 extract for 30 min at 37°C. *s*p6BY was detected by measurement of fluorescence excitation. **(E)** 10 ng *s*p6BY were incubated with 5 μg S10 extract for the times indicated. Band intensities were quantified with AIDA for seven independently performed experiments. Values represent the arithmetic mean ± SD. **(F)** VLPs produced in HEK293T cells transfected with the subgenomic HIV-1 expression plasmid pΔR [11] were isolated, lysed with 0.5% Triton X-100 and incubated with 5 μg S10 extract for 30 min at 37°C. (*) S10 extract, or VLP lysate, was heat-inactivated for 5 min at 95°C prior to incubation. Samples were analyzed by Western blotting. **(G)** VLPs were produced and treated as described in (F) and analyzed for Vpr content.

The observation that CMV-driven expression of p6 cannot be detected by Western blot, yet abundant p6-derived C-terminal epitopes are presented in the context of MHC-I, indicated that newly synthesized p6 undergoes rapid degradation or intracellular modification. To investigate if p6 is subject to proteolytic cleavage, *s*p6 was incubated in cytosolic S10 HeLa cell extract (S10). Western blot analyses revealed a rapid loss of *s*p6 after treatment with S10, indicating first evidence for proteolytic degradation of p6 (Fig 3A). Heat inactivation of S10 prevented this phenomenon, strongly suggesting that the rapid loss of *s*p6 is due to the action of a proteolytic enzyme. Kinetic analyses of *s*p6 in S10 extract under these conditions revealed a rapid, exponential decay with an estimated half-life of about 10 min, which would be characteristic for proteolysis of p6 (Fig 3B and 3C). Similar results were obtained with a *s*p6 peptide labeled with the fluorophore BODIPY FL (*s*p6BY) which allowed sensitive and quantitative detection of p6 directly in SDS gels without staining by antibodies. Coincubation of *s*p6BY with S10 revealed that *s*p6BY is subject to rapid proteolysis (Fig 3D), with a half-life of ~10 min (Fig 3E), comparable to the half-life determined for unmodified *s*p6 by Western blot (Fig 3C). Thus, using *s*p6BY as substrate for *in vitro* degradation enabled us to develop a rapid, sensitive, and quantitative method for screening compounds that interfere with the degradation of *s*p6.

To analyze if *s*p6 degradation is an artifact unique to the synthetic peptide, viral p6 (*v*p6) originating from purified HIV-1 VLPs was incubated with S10. As observed for *s*p6, *v*p6 was degraded by S10 (Fig 3F). Most intriguingly, only *v*p6 disappears after S10 treatment, while all other Gag proteins and the accessory protein Vpr, also encapsidated into virions (Fig 3G), remain completely unchanged. Hence, the effect of S10 appears to be specific for p6, and occurs only with free p6, but not with any of the p6 containing Gag processing intermediates, or even with the Pr55 polyprotein. Heat inactivation of S10 stabilized *v*p6, but heat inactivation of the VLP lysates had no effect, indicating that the p6-degrading activity is present in S10, but not in virions.

**Fig 4:**
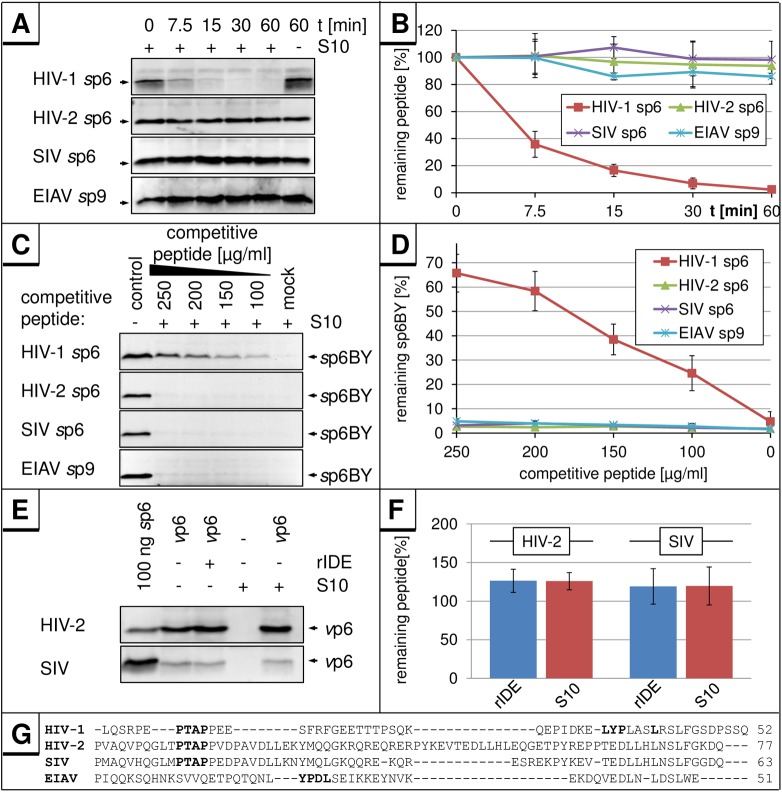
The 51 aa EIAV p9 protein and HIV-2 or SIV p6 are not degraded in S10. **(A)** 400 ng of HIV-2 or SIV *s*p6, or 100 ng of HIV-1 *s*p6, or EIAV *s*p9 were incubated with 5 μg S10 extract for the indicated times at 37°C, and remaining *s*p6 or *s*p9 was detected by Western blot. **(B)** Band intensities were quantified for three independently performed experiments. Values represent the arithmetic mean ± SD. **(C)** 10 ng *s*p6BY were incubated with 5 μg S10 and increasing concentrations of HIV-1, HIV-2 or SIV *s*p6 or EIAV *s*p9 for 30 min at 37°C. *s*p6BY was detected by measurement of fluorescence excitation. **(D)** Band intensities were quantified for three independently performed experiments. Values represent the arithmetic mean ± SD. **(E)** Virions produced in HEK293T cells transfected with expression plasmids pNLgp2/Udel-1 (HIV-2) or pSIV3+ (SIV) were isolated, lysed with 0.5% Triton X-100 and incubated with 5 μg S10 extract or 10 ng rIDE for 30 min at 37°C. **(F)** Band intensities were quantified for three independently performed experiments. Values represent the arithmetic mean ± SD. **(G)** Sequence alignment of p6 peptides from HIV-1, HIV-2, SIV and the EIAV p9 peptide. The sequence of HIV-2 p6 originates from the isolate ROD10, SIV p6 from SIVmac239, and EIAV p9 from the isolate EIAV_Wyoming_ [29].

Next, we asked whether other L-domain containing lentiviral proteins are also subject to this degradation. First, we analyzed the equine infectious anemia virus (EIAV) p9 Gag protein, which represents the L-domain containing EIAV homolog of HIV-1 p6. With a size (51 aa) comparable to HIV-1 p6, it displays little sequence homology to HIV-1 p6 (Fig 4G) [28,29]. In contrast to *s*p6, there was no degradation observed for *s*p9 under conditions in which *s*p6 was rapidly degraded (Fig 4A and 4B). Consistent with that, *s*p9 did not compete with the degradation of *s*p6BY (Fig 4C), while *s*p6 revealed a dose-dependent inhibition of *s*p6BY degradation with an IC_50_ of ~175 μg/ml (30 μM) indicating that *s*p9 does not bind to IDE (Fig 4D).

Similar to the results obtained with *s*p9, we did not observe any degradation of the more closely to HIV-1 related p6 homologs from HIV-2 and SIV (Fig 4A and 4B), nor did these peptides function as competitive inhibitors for the degradation of HIV-1 p6 (Fig 4C and 4D). Similarly, *v*p6 obtained from HIV-2 and SIV virions was inert to S10 treatment (Fig 4E and 4F). In contrast to the results obtained with p6 proteins derived from HIV-2 and SIV, the IDE-mediated degradation of p6 appears to be conserved among different HIV-1 variants. p6-proteins derived from the molecular HIV-1 clones AD8 [30], YU-2C [31,32], NC7 [33], JC16 [33], HXB2 [34], IIIB [34,35], and the HIV-1 field isolates BK132 [36] were tested positive in the S10 *in vitro* degradation assay (S1A Fig). Also, the p6 mutants of HIV-1_NL4-3_ E0A, in which all Glu residues are mutated to Ala [24], S40F, carrying a non-conserved exchange of Ser-40 to Phe [25], the mutant Ser-14 to Asn (S14N), and the ubiquitination-mutant Lys-27/33 to Arg (K27/33R) [14] exhibit *wt* degradation efficiency (S1B Fig). Altogether, the phenomenon observed so far appears to be specific for and conserved within HIV-1 p6.

In summary, the cumulative data demonstrate that a cytosolic enzymatic activity, most likely a protease, is able to specifically degrade p6 from HIV-1 and that this rapid proteolysis only occurs when p6 is released in its mature form from other Gag proteins. This phenomenon appears to be specific for HIV-1 p6 since other L-domain containing retroviral homologs derived from EIAV, SIV or HIV-2 did not succumb to degradation.

### HIV-1 p6 is cleaved by the insulin-degrading enzyme

**Fig 5:**
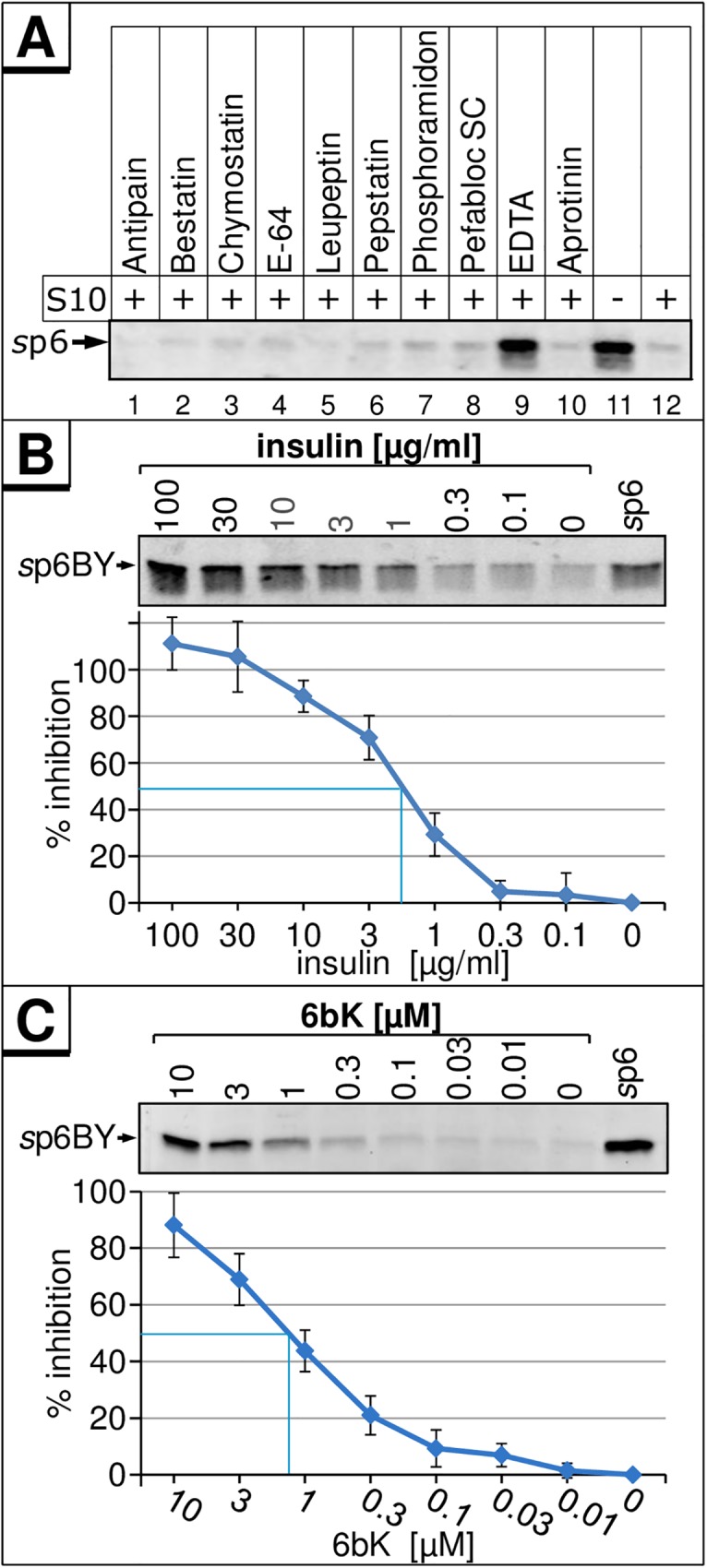
Inhibitors of the metalloprotease IDE block the *in vitro* degradation of *s*p6. **(A)** 100 ng *s*p6 were incubated without or with indicated inhibitors and 5 μg S10 extract for 30 min at 37°C. Remaining p6 was detected by Western blot. 10 ng of *s*p6BY were incubated with 5 μg S10 extract and increasing concentrations of insulin **(B)** or 6bK **(C)** for 30 min at 37°C. *s*p6BY was detected by measurement of fluorescence excitation. Values represent the arithmetic mean ± SD of at least three independent experiments.

To identify the protease responsible for degradation of p6, we utilized inhibitors specific for different classes of proteases. An initial screen revealed that only EDTA could stabilize *s*p6, suggesting that the candidate enzyme is a metalloprotease (Fig 5A).

Screening of the databases MEROPS (http://merops.sanger.ac.uk/, [37]) and Brenda [38] for cytosolic metalloproteases provided a list of candidate proteases which might be responsible for the degradation of p6, including metallo-aminopeptidases (AP), predominantly represented by leucine AP and puromycin-sensitive AP, as well as the endopeptidases Thimetoligopeptidase (THOP1), Nardilysin (NRD), Neurolysin, Endothelin-converting enzyme 1b (ECE1b), and the insulin-degrading enzyme (IDE). Carboxypeptidase activity is generally absent in the cytosol [39,40].

APs could be excluded, as their common inhibitor Bestatin did not stabilize p6 (Fig 5A). ECE1b is reported to be sensitive to Phosphoramidon [41] which failed to stabilize p6 (Fig 5A). NRD only cleaves at dibasic sequences [42] not present in p6. THOP1 exclusively catalyzes the breakdown of peptides shorter than 18 amino acids [43] and could most likely not degrade p6. Thus, only IDE and Neurolysin remained as viable candidates.

Therefore, we investigated the role of IDE in p6 cleavage. IDE is a zinc metalloendoprotease that is ubiquitously expressed in mammals and plays a crucial role in the turnover of peptide hormones such as insulin and glucagon, and of aggregation prone proteins like amyloid-β [44]. We analyzed the impact of the competitive IDE-substrate insulin on *in vitro* degradation of *s*p6. Insulin inhibited p6 degradation in a dose-dependent manner (Fig 5B), and complete inhibition was achieved at concentrations above 30 μg/ml insulin. The concentration for 50% inhibition (IC_50_) was determined at 2 μg/ml (350 nM) insulin which corresponds to about 4-fold molecular excess of insulin compared to *s*p6.

To further confirm that IDE is responsible for the degradation of *s*p6, the recently described IDE- inhibitor 6bK [45] was tested in our *in vitro* degradation assay. Addition of 6bK led to a dose-dependent stabilization of *s*p6 with an IC_50_ of approx. 1.3 μM of 6bK (Fig 5C). Moreover, other known IDE-inhibitors, ATP, NEM, and Bacitracin, also prevented the degradation of p6 *in vitro* (S2 Fig)

To investigate if p6 is also able to interfere with the degradation of a typical IDE-substrate, we analyzed its ability to compete with the *in vitro* degradation of amyloid-β (Aβ) [46]. The effect of increasing amounts of *s*p6 and insulin on the degradation of a FITC-labeled Aβ 1–40 peptide (Aβ-FITC) in S10 was compared demonstrating that *s*p6 inhibits the degradation of Aβ-FITC in a dose-dependent manner with an IC_50_ of 80 μg/ml (14 μM) (S3 Fig). IDE was confirmed as the responsible enzyme as titration of insulin also resulted in a stabilization of Aβ-FITC (S3B Fig).

Thus, the degradation of *s*p6 can be blocked by insulin and 6bK, rendering IDE the most likely protease responsible for the degradation of p6. This notion is supported by the finding that p6 itself can also interfere with IDE-mediated degradation of Aβ (S3 Fig). The fact that p6 is stabilized by excess of insulin also indicates that besides IDE no other proteases in the cytosol contribute to the proteolysis of p6 *in vitro*.

**Fig 6:**
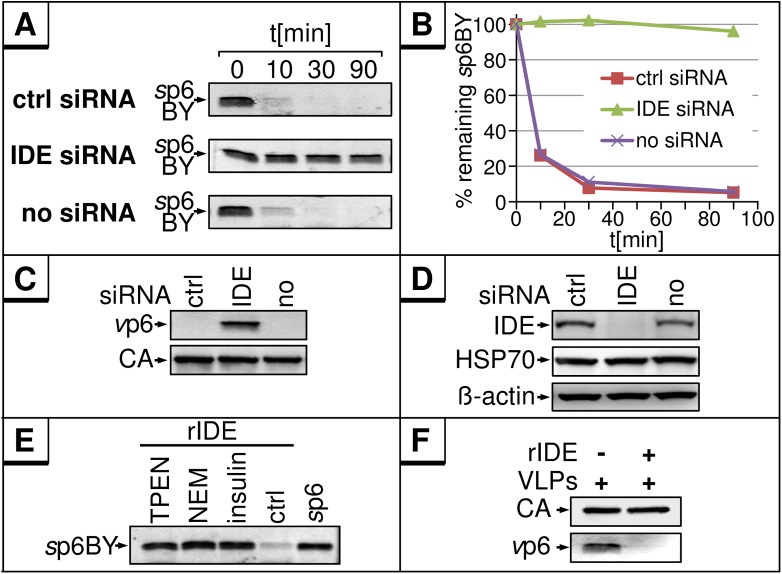
IDE is sufficient and required for the *in vitro* degradation of *s*p6 and *v*p6. **(A)** 10 ng *s*p6BY were incubated with 5 μg S10 extract from HeLa cells, either untransfected or transfected with control or IDE-specific siRNA, for 0, 10, 30 or 90 min at 37°C. **(B)** *s*p6BY was detected by fluorescence emission and quantified. **(C)** VLP lysates from HEK293T cells were incubated with S10 from siRNA treated cells described in A. Viral proteins were detected as described in Fig 1. **(D)** Western blot analyses of the S10 extract used in A and C. The membrane was stained with antibodies specific for IDE, HSP70, and β-actin. **(E)** 10 ng rIDE were incubated with 10 ng *s*p6BY and either TPEN (1 mM), NEM (1 mM), insulin (100 μg/ml) or buffer for 30 min at 37°C. **(F)** VLP lysates were incubated with 10 ng rIDE for 30 min.

To confirm that IDE is primarily responsible for the *in vitro* degradation of p6, lysates were generated from cells wherein IDE was depleted by transfection of an IDE-specific siRNA. *In vitro* exposure of *s*p6BY to IDE-depleted S10 revealed no p6 degradation, while the typical rapid decay of p6 occurred in lysates of *wt* cells or cells transfected with control siRNA (Fig 6A and 6B). The same results were obtained using *v*p6 as substrate, clearly indicating that IDE is sufficient and required for degradation of p6 (Fig 6C). The efficiency of the IDE knock down was confirmed by Western blotting (Fig 6D). Additionally, *s*p6 was degraded by recombinant IDE (rIDE) (Fig 6E). Also, the inhibitors TPEN, a Zn^++^-specific chelator, NEM or insulin stabilized *s*p6 in the presence of rIDE demonstrating the degradation is not due to contaminating proteases in the enzyme preparation. Moreover, rIDE could also cleave *v*p6 from HIV-1 (Fig 6F), whereas it had no effect on other HIV-1 proteins like CA (Fig 6F). Consistent with the results obtained with S10, rIDE did also not degrade p6 in case of HIV-2 or SIV (Fig 4E and 4F).

To transfer the *in vitro* data to a physiologically more relevant context, e.g. to analyze *in cellulo* the relevance of the p6-IDE-interaction, an IDE KO HeLa TZM-bl cell line was generated by a CRISPR/Cas9 approach. HeLa TZM-bl *wt* and IDE KO cells were transiently transfected with a CMV-driven p6 expression plasmid or the empty vector pcDNA. Although equal amounts of lysate were analyzed by Western blotting, p6-protein was only detectable in HeLa TZM-bl IDE KO cells, but not in *wt* cells (S4 Fig). This further supports our assumption that IDE is the most prominent proteolytic activity influencing the steady state level of intracellular p6.

**Fig 7:**
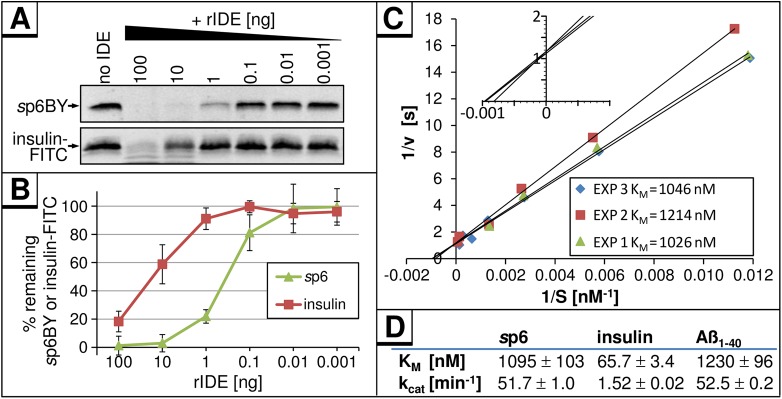
*s*p6 is an up to 100-fold better IDE-substrate than insulin. **(A)** 10 ng of *s*p6BY or insulin-FITC were incubated with indicated amounts of rIDE for 30 min at 37°C. Remaining amounts of *s*p6BY and insulin-FITC were detected by measurement of fluorescence excitation. **(B)** Results of four independently performed experiments. Values represent the arithmetic mean ± SD. **(C)** Increasing concentrations of *s*p6BY were incubated with rIDE for 10 min at 37°C. The velocities were calculated from the degradation of *s*p6BY and normalized for the amount of rIDE. Data points represent values from three independent experiments in a double-reciprocal Lineweaver-Burk plot. The inset shows a magnification of the intersections of the regression lines with the axes. **(D)** Comparison of K_M_ and v_max_ values for *s*p6BY and IDE as determined in (C) to those of IDE and insulin or Aβ as reported [47].

To compare the efficiencies of IDE-mediated cleavage of p6 and insulin, rIDE was added in decreasing amounts to 10 ng of each substrate, *s*p6BY or FITC-labeled insulin (insulin-FITC) (Fig 7A). Quantification of remaining IDE-substrates revealed that 1 ng rIDE was sufficient to degrade 80% of *s*p6 within 30 min (Fig 7B), while 100-fold more rIDE was required to cleave insulin-FITC with the same efficiency.

The turnover of *s*p6BY by rIDE had a K_M_ of 1.1 μM (Fig 7C) which is comparable to the K_M_ of Aβ (1.2 μM), but about 16-fold higher than that reported previously for insulin-degradation using purified mammalian IDE (66 nM, Fig 7D) [47]. The k_cat_ for IDE-mediated degradation of *s*p6BY was calculated to be 51.7 min^-1^ (Fig 7D) which was comparable to k_cat_ previously determined for Aβ (52.5 min^-1^), but 34-fold higher than in the case of insulin (1.5 min^-1^, Fig 7D) [47].

In aggregate, these results indicated that IDE is sufficient and required to specifically degrade p6 *in vitro*, and that this lentiviral peptide represents a 30- to 100-fold better substrate for IDE than the evolutionary substrate insulin.

### Multiplication of the PTAPPA L-domain motifs prevents the IDE-mediated degradation of p6

**Fig 8:**
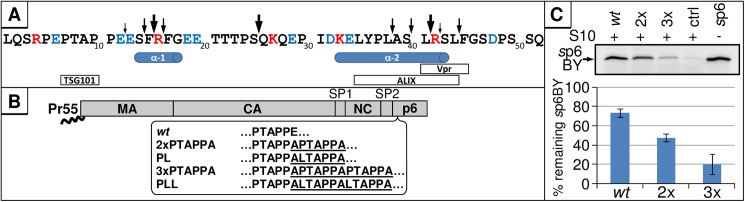
Generation of stable p6 mutants. **(A)** 100 ng *s*p6 were incubated with 10 ng rIDE for 1, 5, 10, 30 or 60 min at 37°C. Reactions were stopped by adjusting the samples to 0.3% (w/v) TFE and subsequently analyzed by mass spectrometry. Arrows above the primary sequence represent the detected cleavage sites, and initial and secondary cleavage sites are indicated as big or small arrows, respectively. Red font indicates positively charged, and blue negatively charged residues. Previously identified α-helices and binding motifs are depicted below the primary sequence. **(B)** p6 mutants that encode multiple PTAPPA- or LTAPPA-motifs were generated. The introduced amino acids are underlined. **(C)** 10 ng of *s*p6BY were incubated with 250 μg/ml *s*p6 *wt*, 2xPTAPPA, 3xPTAPPA or only buffer and 5 μg S10 for 30 min at 37°C. *s*p6BY was detected by fluorescence emission and quantified. Values represent the arithmetic mean ± SD of four independent experiments.

To investigate the biological consequence of p6-degradation, we generated an IDE-insensitive p6-mutant. No general recognition sites or other consensus sequences have been reported for standard IDE substrates such as insulin or glucagon [48]. To reveal the cleavage sites in p6, we performed mass spectrometry analysis of p6 fragments generated by IDE. Ten cleavage sites were identified, of which three major cleavages appear to occur shortly after degradation of p6 is initiated. The primary cut occurs between S25/Q26, while two secondary cleavage sites are present between F15/R16 and L41/R42. During later stages of degradation, 7 additional auxiliary cleavage sites were observed (Fig 8A).

**Fig 9:**
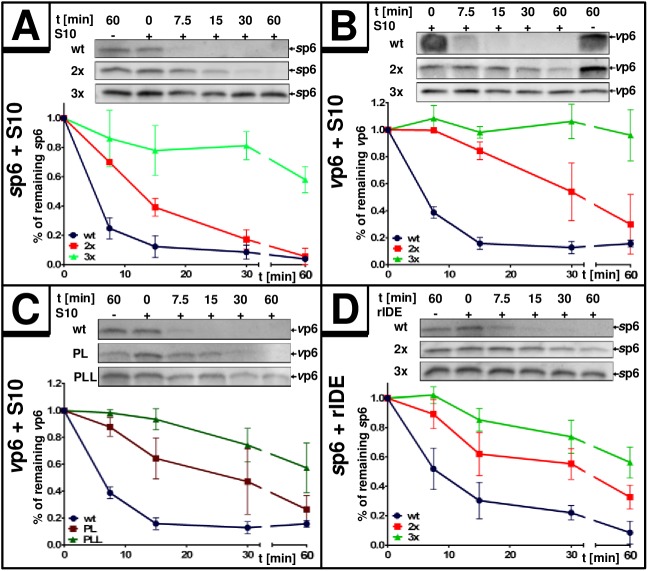
Multiplication of PTAPPA-motifs stabilizes p6. 20 ng of *s*p6 or 30 ng of *v*p6 were incubated either with 5 μg S10 extract **(A/B/C)** or 2 ng of rIDE **(D)** for up to 60 min. Degradation efficiency was quantified *via* densitometric analyses of Western blots. Values represent the arithmetic mean ± SD of at least 3 independent experiments for each setting.

In order to establish a mutant version of p6 that cannot be degraded by IDE, we modified typical features of an IDE substrate within the p6 sequence. First, we altered the N-terminus as the suspected primary binding site for IDE [48] by duplication of the N-terminal PTAP-motif, a mutation that is frequently observed in clinical HIV-isolates as it augments virus maturation under antiviral treatment [49,50]. To mimic these natural N-terminal alterations, we generated a p6 mutant containing two PTAPPA domains (further referred to as 2x, Fig 8B) and tested its stability by *in vitro* degradation assays using S10 and rIDE combined with synthetic or virally-produced substrates of the 2x mutant version. Depending on the experimental setting, the 2x mutant had a half-life of up to 30 minutes compared to the 7 min-half-life of *wt* p6 (Fig 9A, 9B and 9D). Then, a p6 variant containing three PTAPPA-motifs (further referred to as 3x) was generated and tested under the same conditions. The 3x mutant even showed greater stability with a half-life of more than 60 minutes established for either *s*p6 or *v*p6 as substrate (Fig 9A, 9B and 9D).

In general, PTAPPA-motifs represent binding sites for the ESCRT-component TSG101 [51]. To rule out a possible contribution of TSG101 present in S10 to the enhanced stability of the 2x and 3x mutants, we altered the PTAPPA-motifs of the 2x and 3x mutants to LTAPPA (further referred to as PL and PLL, Fig 8B), a mutation which abolishes interaction with TSG101 [52]. The PL and PLL mutants contain only one functional PTAP motif and should exhibit *wt*-like interaction with TSG101. However, the degradation kinetics of PL/PLL *v*p6 were comparable to their 2x and 3x counterparts, indicating that TSG101 is not involved in the stabilization of p6 upon introduction of PTAPPA multiplications (Fig 9C).

Competition experiments were performed to investigate if multiplication of the PTAPPA-motif interferes with substrate recognition of p6 by IDE. The capacity of unlabeled *s*p6, either as *wt* or as the 2x and 3x PTAPPA mutants to interfere with the degradation of *s*p6BY *in vitro* was analyzed. Quantification of the remaining *s*p6BY revealed diminished efficiency for the 2x and 3x mutants to interfere with degradation of *s*p6BY (Fig 8C) indicating that multiplication of PTAPPA motifs abrogates the ability of those p6 mutants to bind to IDE.

### Multiplication of the PTAPPA motif does not affect the structure of p6

To investigate whether the duplication or triplication of the PTAPPA motif of p6 had any effects on the positions and extent of secondary structure of the mutant proteins, NMR spectra of the 2x and 3x mutants dissolved in H_2_O-TFE-D_2_ (1:1) were obtained. It has been shown experimentally that α-proton chemical shifts greater than 0.1 ppm relative to the random coil values are qualitative indicators of protein secondary structure. A minimum of four adjacent residues with an upfield shift is indicative of an α-helix, whereas β-sheets require a minimum of three residues with downfield shifts [53]. After spectral assignment, chemical shift index (CSI) plots (S5 Fig) that indicate the location of the α-helical structure were calculated. In both cases of the mutant proteins, the localisation and extent of α-helical structure appears to be the same as for the *wt* protein [18,54] (S5 Fig), consistent with the fact that the additional PTAPPA motif(s) locate to a region where p6 generally exhibits random structure (S5 Fig) [18]. All positive values for N-terminal residues adjacent to proline residues arise from an inherent effect of proline and not out of a structural perturbation, as described in [55].

### IDE-insensitive mutants of p6 show normal virus release, Gag processing and Vpr encapsidation

**Fig 10:**
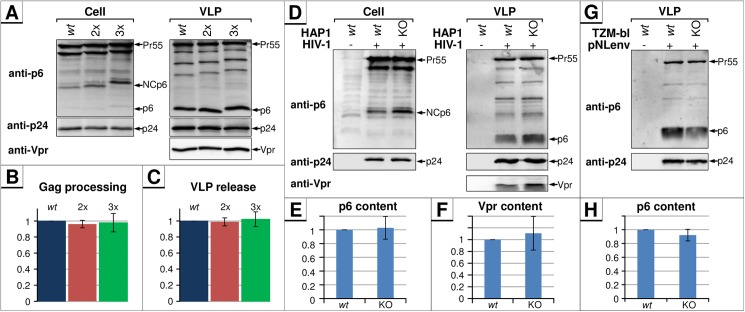
The IDE-p6-interaction has no effect on Gag-processing and virus release. **(A)** HEK293T cells were transfected with pΔR plasmids encoding for either *wt* Gag, or the p6 mutants 2xPTAPPA or 3xPTAPPA. Cells were lysed and VLPs were purified and subsequently analyzed by Western blot. Noteworthy, Gag processing and virus release of 2x and 3x PTAPPA mutants were comparable to that of the *wt*, only the apparent molecular weight of p6 and the NCp6 processing intermediate increased by PTAPPA multiplication. **(B)** The rate of Gag processing was estimated by calculating the ratio of p24 vs. Pr55 detected in released VLPs. Bars represent mean values of three independent experiments ± SD. **(C)** Efficiency of virus release was calculated as the ratio of Gag (Pr55 and p24) present in the virus pellet relative to the total amount of Gag detected in cells and released VLPs. Bars represent mean values of three independent experiments ± SD. Both Gag processing and virus release for the *wt* were set to 1. **(D)** HAP1 *wt* cells and HAP1 IDE knock out cells were infected with VSV-G-pseudotyped *wt* HIV-1 particles. 2 days post-infection, cell and virus-fractions were harvested and analyzed by Western blot for viral proteins. Band intensities of virus-associated p6 **(E)** and Vpr **(F)** were quantified and normalized for p24 signals. Bars represent mean values of three independent experiments ± SD. **(G)** HeLa TZM-bl *wt* and IDE KO cells were transiently transfected with pNLenv1 and virus and cell fractions were analyzed by Western blot. **(H)** Band intensities of virus-associated p6 were quantified as described in (E).

The PTAP motif generally regulates virus maturation and budding [56]. To investigate if PTAPPA insertion influences those late processes in the viral life cycle, HEK293T cells were transfected with pΔR encoding either p6 *wt*, or the 2x and 3x mutants, and Western blot analyses of cell and VLP fractions were performed. Intriguingly, multiplication of PTAPPA-motifs, although preventing IDE-mediated degradation, did not affect the L-domain function of p6 in virus release and Gag processing (Fig 10A–10C). Furthermore, encapsidation of Vpr into VLPs was also not affected (Fig 10A).

This notion was further confirmed by analyzing virus production in *wt* versus IDE knock-out cells (IDE-KO). Therefore, CRISPR/Cas9-edited haploid HAP1 cells [57] were infected with VSV-G-pseudotyped HIV-1 *wt* particles. The pattern of viral proteins was almost identical between *wt* and IDE-KO settings of cell and virus fractions, demonstrating that virus release and maturation do not depend on IDE-activity (Fig 10D). Additionally, the amounts of incorporated Vpr as well as the abundance of mature p6 in particles produced by IDE-KO cells were similar to those of the parental cells (Fig 10E and 10F). Similar results in terms of Gag maturation and the amount of virus-associated p6 were obtained by studying VLP release from HeLa TZM-bl *wt* and IDE KO cells after transient transfection with the pNL*env*1 HIV-1 expression construct (Fig 10G and 10H). Altogether, these results indicate that the IDE-mediated degradation of p6 is not relevant during the late steps of HIV-1 replication.

In summary, the introduction of multiple PTAPPA-motifs does not affect the local backbone folding of p6, but might interfere with IDE binding and leads to dose-dependent stabilization of p6. Moreover, the p6-IDE-interaction does not seem to be required for the established functions of p6 as part of Pr55, e.g. virus release, Gag-processing, and Vpr-incorporation. Altogether, the cumulative results indicate that IDE activity does not influence L-domain function and that L-domain function does not require the degradation of p6.

### The stability of p6 correlates inversely with the replication capacity of HIV-1 and the responsiveness to insulin and 6bK in primary T cells derived from activated PBMCs

**Fig 11:**
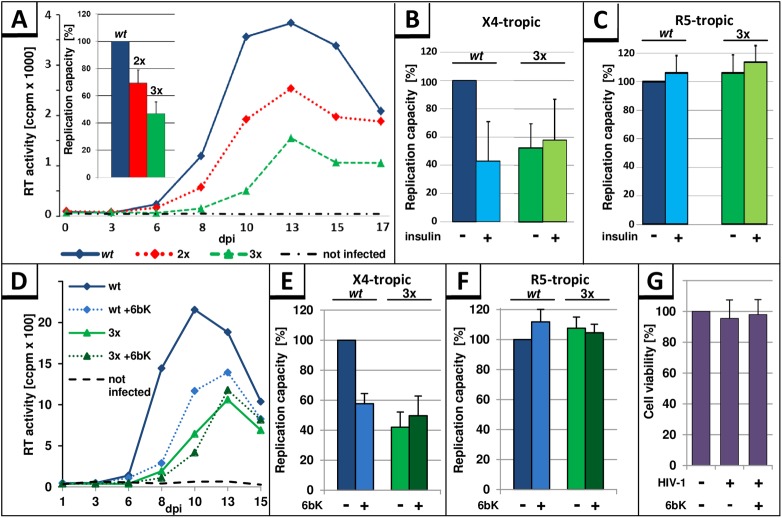
The stability of p6 correlates inversely with the replication capacity of HIV-1 and sensitivity to insulin in X4-tropic replication. **(A**) A representative replication profile of HIV-1_NL4-3_ variants is shown for PHA-IL2-stimulated PBMCs, infected with *wt*, 2xPTAPPA (2x), 3xPTAPPA (3x) (30 pg p24, MOI 10^−4^) or mock infected, and replication was assessed by quantification of the virus-associated reverse transcriptase (RT) activity contained in cell culture supernatant collected on the indicated days post infection (dpi). The replication capacity of X4-tropic HIV-1_NL4-3_ *wt*, 2xPTAPPA or 3xPTAPPA following infection of PHA-IL2-stimulated PBMCs from 6 different donors was assessed by calculating the area under the curve (AUC) from each individual replication profile. The replication capacity of HIV-1_NL4-3_ *wt* in each experiment was set to 100%. Error bars, ± SD (Inset). (**B**) Replication capacity of X4-tropic HIV-1_NL4-3_ *wt* or 3xPTAPPA with or without permanent treatment with 50 μg/ml insulin following infection of PHA-IL2-stimulated PBMCs from 3 different donors. The replication capacity of HIV-1_NL4-3_ *wt* from each experiment was set to 100%. Error bars, ± SD. (**C**) Replication capacity of R5-tropic HIV-1_NL4-3_ *wt* or 3xPTAPPA with or without permanent treatment with 50 μg/ml insulin following infection of PHA-IL2-stimulated PBMCs from 3 different donors. The replication capacity of HIV-1_NL4-3_ *wt* from each experiment was set to 100%. Error bars, ± SD. **(D)** PHA-IL2-stimulated PBMCs were infected with HIV-1_NL4-3_ *wt* or 3xPTAPPA and permanently treated with 10 μM 6bK, or were left untreated. Replication capacities were determined as described in (B) for PHA/IL-2-stimulated PBMCs from 3 different donors following infection with X4 **(E)** or R5 tropic **(F)** viruses. **(G)** Cell viability was assessed by water-soluble tetrazolium salt assay on the last day of replication study.

Next, we analyzed if stabilization of p6 by PTAPPA insertions affects HIV-1 replication. Activated human PBMCs were inoculated with standardized amounts of X4-tropic HIV-1_NL4-3_, either *wt*, the 2x, or 3xPTAPPA mutants. Cell culture supernatants were collected on the indicated days post infection (dpi) and analyzed for release of virus particles by measuring the virus associated reverse transcriptase (RT) activity. The representative replication profile in Fig 11A shows that insertion of PTAPPA-motifs inhibits HIV-1 replication in a dose-dependent manner. Since the replication of HIV-1 in PBMCs generally exhibits donor-dependent differences, the area under the curve (AUC) representing the replication capacity in PBMCs was determined. Replication profiles of *wt* compared to p6 mutants in PBMCs derived from 6 different donors revealed up to 30% reduction for the 2xPTAPPA mutant, and up to 50% reduction for 3xPTAPPA mutant in replication capacity (Fig 11A, inset).

We also examined the effect of insulin on the replication of HIV-1 *wt*, compared to the 3x mutant in PBMC cultures. Evaluation of HIV-1 replication following infection of PBMCs from 3 different donors showed that continuous treatment of cells with 50 μg/ml insulin leads to ~50% reduction of HIV-1 replication (Fig 11B). In order to clarify whether this effect of insulin on the HIV-1 replication is specifically caused by the stabilization of p6, PBMC cultures were infected with the 3xPTAPPA variant of HIV-1_NL4-3_ virus and treated with insulin. The 3xPTAPPA mutant was insensitive to insulin treatment, supporting the assumption that the replication capacity of HIV-1 correlates inversely with the stability of p6 (Fig 11B).

Upon infection of PBMCs with R5-tropic HIV-1_NL4-3_, which is isogenic to its X4-tropic counterpart, except the 005pf135 V3 loop region in Env [58], neither *wt* nor 3xPTAPPA mutant revealed any differences in the replication capacity of HIV-1 or any response to insulin treatment (Fig 11C), indicating that multiplication of PTAPPA *per se* or insulin treatment has no unspecific negative impact on HIV-1, since it only occurs in X4-tropic virus replication.

In order to further confirm the role of IDE in replication of X4-tropic HIV-1, the effect of the pharmaceutical IDE-inhibitor 6bK [45] was tested. Consistent with the notion of an inhibitory function of stable p6, permanent treatment with the IDE inhibitor 6bK reduced the replication of X4-tropic HIV-1 *wt* (Fig 11D and 11E). Quantification of the replication capacity revealed a 50% reduction in the replication capacity of the *wt* under 6bK treatment (Fig 11D and 11E), while cell viability was not affected (Fig 11G). However, the IDE-insensitive 3xPTAPPA mutant shows no further reduction in the replication capacity following treatment with 6bK, which underlines our hypothesis about an inverse correlation between the replication capacity of X4-tropic HIV-1 and the IDE-mediated degradation of its p6 protein. In contrast, 6bK had no influence on the replication capacity of R5-tropic HIV-1 variants, for both the *wt* and the 3xPTAPPA (Fig 11F). To test if the inverse correlation between the replication capacity of X4-tropic HIV-1 and the IDE-mediated degradation of p6 is a general phenomenon and not specific for HIV-1_NL4-3_, we analyzed the influence of permanent treatment with insulin or 6bK on the replication capacity of the X4-tropic field isolate HIV-1 BK132 [36] (S6 Fig). Although there is only an 88.46% sequence identity between these two HIV-1 p6 variants, the p6 protein of BK132 is susceptible to IDE degradation (S1A Fig). In consistency, the same impact of insulin and 6bK on replication capacity was observed for HIV-1 BK132 (S6 Fig) as it was observed for HIV-1_NL4-3_ (Fig 11). In summary, the inhibitory effect of 6bK on the HIV-1 replication capacity occurs in a range similar to that of the 3xPTAPPA mutant, or for HIV-1 *wt* under insulin treatment.

These findings altogether indicate that under certain conditions, that seem to be dependent on the presence of X4 tropic Env, the replication capacity of HIV-1 inversely correlates with the stability of p6.

## Discussion

All functions of p6 have been described in the context of p6 as the C-terminal part of the Gag polyprotein. However, p6 is abundant in virions and, if it is there equimolar to other Gag proteins, it should account for up to 5000 molecules per incoming virus to be present in the target cell upon virus entry. The observation that p6 is found only in released virions prompted us to investigate the fate and function of p6 as mature protein. In the present study, we have characterized HIV-1 p6 as the first viral IDE-substrate. Although p6 is catalyzed by IDE at rates which are comparable to the proteolysis of Aβ, it is ~100-fold more efficiently degraded by IDE than its eponymous substrate insulin. So far we have not been able to completely unravel the biological role of this phenomenon. Nevertheless, it appears that the rapid degradation of p6 by IDE, following its entry into the cell, represents a crucial event to achieve optimal replication capacity of HIV-1 under certain conditions.

Our initial attempts to study the function of free p6 in virus producing cells demonstrated that free p6 exists predominantly in virions, but is hardly detectable intracellularly [18]. Attempts to express p6 from a codon-optimized transgene failed in *wt* HeLa cells, consistent with earlier reports describing unsuccessful attempts to express free p6 in HEK 293T cells [10]. However, a p6-YFP fusion was readily expressed from the same expression system. Most intriguingly, the MHC-I presentation of an SL epitope introduced at the C-terminus of p6 provided strong evidence that p6 in its mature form must have been expressed, further supporting our assumption that the protein is rapidly degraded shortly after its translation.

By establishing an *in vitro* degradation assay using *s*p6, *s*p6BY, and *v*p6 as substrates and S10 as source of enzymatic activity, we could demonstrate that p6 undergoes rapid decay with a half-life of ~10 min or less in *ex vivo* cytoplasmic environments. Moreover, by use of IDE-specific inhibitors and siRNA-mediated knock down of IDE, we could identify IDE as the p6-degrading activity and demonstrate that besides IDE no other cellular factors or proteases contribute to, or are required for the rapid decay of p6 in the ex v*ivo* S10 degradation system. Thus, it is conceivable to assume that in context of a virus infecting a target cell, p6 is also degraded by IDE shortly after virus entry. Thus, the following *in vitro* and *in cellulo* data indicate that IDE is sufficient and required for the degradation of p6: (1) degradation can be inhibited with 6bK or insulin, (2) as well as with siRNA-mediated IDE depletion in S10. Furthermore, (3) recombinant IDE efficiently degrades p6 *in vitro*. And, finally, (4) expression and detection of p6 from a transgene is only successful in IDE KO cells. Nevertheless, we cannot entirely exclude the existence of other factors which might contribute to the clearance of p6 *in vivo*.

Mass spectrometric analyses of p6-fragments after *in vitro* digestion with rIDE revealed sequential cleavage of p6 resulting in a complex mixture of degradation products, a degradation cascade that has been also observed for IDE-mediated proteolysis of insulin [59]. By expression of a p6-SL fusion protein, we could show that p6-derived epitopes efficiently enter the MHC-I pathway. IDE was reported to regulate the stability of melanoma-associated tumor antigen A3 (MAGE-A3), and in this process is also responsible for processing of a MAGE A3 derived MHC-I epitope [60]. Thus, p6 might be processed by IDE to generate MHC-I antigenic peptides. Until now, the only described p6-derived MHC-I epitope is ^33^KELYPLTSL^41^, which is presented by HLA-B60 to cytotoxic T cells [61]. The C-terminus of this antigenic peptide is identical with one of the prominent IDE cleavage sites detected in our study. It is conceivable that this immunodominant epitope of p6 originates from IDE-mediated processing of p6 and that this phenomenon should also contribute to immune recognition of HIV-1^+^ cells *in vivo*.

The specificity by which IDE recognizes its substrates primarily originates from IDE’s composition of an N- and a C-terminal domain which enclose the catalytic chamber just large enough to accommodate its natural 51 amino acid substrate insulin [48]. Thus, for stabilization of p6, multiplication of the N-terminal PTAPPA motif was appealing since natural PTAP-duplications have been described to emerge frequently in patients [62,63,64]. Insertion of one or two PTAPPA sequences increases the size of p6 from 52 to 58 or 64 aa, respectively, shifting the protein sequence towards the size limit of a typical IDE-substrate. Consistent with that notion, our competition degradation assay indicates a decreased binding of stable p6 variants to IDE, rather than normal binding concomitant with slower degradation. Alternative explanations for the stabilization of p6 by insertion of PTAPPA motifs, for example by increased binding of TSG101 or other cellular factors to p6, could be excluded by efficient degradation of *s*p6 using only rIDE or by studying the TSG101 insensitive mutant PLL. To exclude that multiple sterically demanding Pro residues in the region adjacent to the N-terminal α-helix of p6 influence the extent and position of secondary structure of the protein, the secondary structure was determined. The NMR data revealed that duplication or triplication of the PTAPPA motif does not influence the position or extent of the α-helical structures in p6. Both mutants (2x and 3xPTAPPA) exhibit structures resembling that of the *wt* protein. Thus, it is unlikely that the observed stabilization of the 3xPTAPPA p6 mutant could be explained by changes in the secondary structure.

The size limit imposed by the catalytic chamber of IDE also explains the phenomenon that p6 in the context of the Gag intermediate processing products e.g. NCp6, Pr55, or even of our p6-YFP fusion, does not undergo IDE-mediated proteolysis. This particular feature of IDE might be exploited by HIV-1 to assure that only mature p6, but none of the other p6 containing Gag proteins, is removed. In the same line, the p6 proteins of HIV-2 and SIV with 77 and 63 aa in length, respectively, are also insensitive towards IDE, most likely because of their oversize for IDE. Nevertheless, the degradation of HIV-1 p6 does not solely originate from its small size, but occurs in a sequence and probably in a structure-dependent manner, inasmuch as the similar sized EIAV p9 protein, with 51 aa in length, is resistant to IDE. Altogether, we have tested p6-proteins from 12 HIV-1 isolates and mutants as a substrate for IDE, indicating that degradation of p6 represents a phylogenetically conserved phenomenon within HIV-1. However, it cannot be entirely excluded that there are also HIV-1 p6-variants that are less or not susceptible to IDE-mediated degradation.

In order to get a very first insight into the biological function of the phenomenon described herein, the replication capacities of the 2x and 3x PTAPPA mutants were studied in the context of HIV_NL4-3_ in activated PBMCs. In case of X4-tropic HIV-1_NL4-3_, a negative correlation of the p6-stability and the replication capacity was observed. One possible explanation for the reduced replication capacity of the 3xPTAPPA mutant could be that multiplication of the PTAP-motif interferes with the late steps of virus replication leading to the production of defective virus particles. However, we did not observe any differences in Gag maturation, virus release, and Vpr incorporation for *wt* and the 2x and 3x PTAPPA mutants. Similar results were obtained by analyzing virions produced by IDE knock out cells. In addition, the replication capacity of the R5-topic 2x and 3x mutants was similar to *wt*, demonstrating that PTAPPA multiplication has no general negative impact on HIV-1 replication.

To further analyze the function of IDE in virus replication, the impact of the IDE-inhibitors insulin and 6bK was analyzed. Permanent treatment of infected PBMCs with insulin revealed a 50% decrease in HIV-1 *wt* replication, whereas it had no effect on the replication of the 3xPTAPPA mutant, harboring an IDE-insensitive p6. In contrast, replication of an R5-tropic variant of HIV-1_NL4-3_, which only differs in 7 aa in the V3-loop of Env [58], was identical to that of the respective 3xPTAPPA mutant and also insensitive to exogenous insulin. This indicates that the importance of IDE for replication is somehow correlated to the nature of the cell tropism-determining region of Env. Inhibitory effects of insulin on HIV-1 replication in human primary cells have been reported earlier in a range comparable to our phenomenon for the isolate HIV-1 IIIB [65]. Consistent with this, we found that the p6-protein of HIV-1 IIIB is degraded in S10. Even more strikingly, treatment of activated PBMCs with the pharmacological IDE-inhibitor 6bK showed very similar effects on the replication capacity of X4-tropic viruses as the treatment with insulin and had, again, no effect on R5-tropic virus replication. Of note, and in contrast to the permanent insulin treatment, unspecific off- target effects of 6bK can be excluded at the concentration (10 μM) used in our experiments, as it was shown previously that also very high concentrations of 6bK (>100 μM) had no side effects *in vivo* [45]. The fact that the replication capacity of the X4-tropic field isolate HIV-1 BK132 is reduced by insulin or 6bK in a similar manner as it has been observed for X4-tropic HIV_NL4-3_ (S6 Fig) is indicative for our assumption that the phenomenon is phylogenetically conserved among HIV-1. In summary, our first biological analyses show that the stability of p6 inversely affects the replication capacity of HIV-1 in an Env-dependent manner. However, until now we could only observe this phenomenon following infection of activated PBMCs, but not in permanent T cell lines (e.g. Jurkat, CEM) and only if cells were infected with relatively low MOI of 10^−4^, which however reflects the range previously reported for infection events occurring naturally in the peripheral blood of HIV-1 patients [66,67,68]. However, the mechanism how the IDE-mediated degradation of p6 affects the replication cycle of HIV-1 remains elusive so far. It might be possible that clearance of p6 after virus entry is important to release Vpr [8,69]. Stable p6-Vpr complexes might prevent the import of the pre-integration complex (PIC) into the nucleus by masking Vpr that is otherwise essential for interaction of PIC with the nuclear pore complex [70].

IDE was not detected in HIV-1 particles [71] and *v*p6 remains stable in *ex vivo* incubated purified virus fractions. Therefore, it can be assumed that IDE must be actively excluded from encapsidation into budding virions, and it should be in the interest of the virus to initiate the degradation of p6 only at the point of virus entry into the host cell. Given that about 10^10^ virions are produced and disintegrated per day [72], and each virion contains 2000–5000 p6 molecules, it should result in a concentration of up to 100 pM of mature p6 in the peripheral blood of an HIV-1^+^ high viraemic individual, assuming that cell- and virus-free version of p6 does not succumb to proteolysis by IDE present in the extracellular space. By competing with the degradation of natural IDE substrates like insulin or Aβ, p6 might have the potential to interfere with insulin processing and clearance of Aβ. In an IDE-KO mouse model, such “hypofunction” of IDE in relation to intracellular insulin and Aβ has been shown to increase cerebral accumulation of endogenous Aβ, as well as cause hyperinsulinemia and glucose intolerance [73]. Hyperinsulinemia / insulin resistance, glucose intolerance, and cognitive disorders related to Alzheimer disease are common features of patients with chronic HIV infection [74,75]. It is plausible that the strong competition of p6 for IDE could contribute to the metabolic disorders associated with insulin resistance as well as cognitive defects associated with Aβ aggregation in HIV patients.

## Material and methods

### Ethics statement

Ethics approval for the sample collection has been obtained from the Ethics Committee of the Medical Faculty of the Friedrich-Alexander Universität Erlangen-Nürnberg. Completely anonymized blood samples were obtained from the blood bank Institute of Transfusion Medicine (Suhl, Germany).

### HIV isolates and expression plasmids

The NL4-3 [76] derived expression constructs pNLenv1 [17], pΔR [11], and the R5-tropic derivative carrying the 005pf135 V3 loop region in Env [58] have been previously described. Additional PTAPPA motifs in p6 were inserted by multiple rounds of site-directed mutagenesis (QuikChange Lightning, Stratagene) using a pair of complementary primers cca aca gcc cca cca gcc cca aca gcc cca cca gcc gag agc ttc agg ttt and aaa cct gaa gct ctc ggc tgg tgg ggc tgt tgg ggc tgg tgg ggc tgt tgg. Introduction of LTAPPA motifs was carried out by mutagenesis using the plasmids coding for additional PTAPPA motifs as template and primer pair gcc cca cca gcc ctg aca gcc cca cca and tgg tgg ggc tgt cag ggc tgg tgg ggc. The plasmids for expression of HIV-2 pSIV3+ [77] and pNLgp2env1-UNY, an *env*-deleted version of pNLgp2/Udel-1 [78] as well as the HIV-1 clones YU-2C [31,32], NC7 [33], JC16 [33] and pAD8env1-Udel1 [79], a derivative of AD8 [30], have been described earlier. The molecular clone HXB2 [34], and the field isolates IIIB [34,35] and BK132 [36], respectively, were obtained by collecting cell culture supernatant from infected CEMx174 cells [80]. The p6-mutant S40F has been described earlier [25,81]. pNLenv1 with the mutations S14N and K27/33R within p6 were generated by mutagenesis using complementary primers ccc acc aga aga gaa ctt cag gtt tgg gg and ccc caa acc tga agt tct ctt ctg gtg gg (S14N) or ccc tct cag agg cag gag ccg ata gac agg gaa ctg tat and ata cag ttc cct gtc tat cgg ctc ctg cct ctg aga ggg (K27/33R), respectively. Sequence identity of the amino acid sequences of the HIV-1 p6 variants NL4-3 and BK132 was determined using an online tool [82].

### Cloning of CMV-driven expression plasmids

For cloning of CMV-promoter driven p6 expression plasmids, a nucleotide sequence codon-optimized for human cells encoding the amino acid sequence derived from NL4-3 p6 was designed *in silico* by using JCat (http://www.jcat.de/, [83]). An N-terminal Met as well as a 5’ BamHI and a 3’ EcoRI restriction site were added. Corresponding overlapping DNA oligonucleotides were designed by *Assembly PCR Oligo Maker* [84], purchased from Biomers (Ulm, Germany), and a continuous DNA fragment was generated thereof by *assembly*-PCR according to previously established protocols [85,86] using the Vent DNA polymerase (New England Biolabs). The resulting PCR product was ligated into pcDNA 3.1 (Invitrogen) using BamHI, EcoRI (New England Biolabs), and T4 ligase (NEB). A p6-YFP fusion was established by overlap-PCR with the codon-optimized *p6* gene and *eYFP* as templates and cloned into pcDNA 3.1 (p6-YFP). Additionally, plasmids were established in which an AU1-tag or the SIINFEKL epitope (SL) followed by stop codons were added at the C-terminus of p6 (p6-AU1 or p6-SL, respectively) by site-directed mutagenesis (QuikChange Lightning, Agilent Technologies). The plasmid driving the expression of Vpu-AU1 has been described previously [21,87].

### Transfection and cell culture

HeLa SS6 [88], HeLa TZM-bl cells (both cell lines kindly provided by Karin Metzner, Institute of Virology, Friedrich-Alexander-University Erlangen-Nürnberg, Erlangen, Germany), and HEK293T (ATCC CRL-1573) were cultivated in DMEM supplemented with 10% (v/v) inactivated fetal calf serum (FCS), 2 mM L-glutamine, 100 U/ml penicillin, and 100 μg/ml streptomycin. HeLa-K^b^ cells [26], kindly provided by Ian A. York, Department of Pathology, University of Massachusetts Medical Center, Worcester, were maintained in DMEM with additional 1 mg/ml geneticin. CEMx174 cells [80] (kindly provided by Karin Metzner, Institute of Virology) were cultured in RPMI supplemented with 10% (v/v) inactivated fetal calf serum (FCS), 2 mM L-glutamine, 100 U/ml penicillin, and 100 μg/ml streptomycin. HAP1 *wt* and IDE knock out clones 10 and 12 were obtained from Haplogen and were cultivated in IMDM containing antibiotics and FCS. All cell culture reagents and media were purchased from Gibco. Confluent monolayers of HeLa and HEK293T cells were transfected with Lipofectamine 2000 (Invitrogen) using the manufacturer’s protocol. For Western blot analyses cells were lysed in RIPA buffer (150 mM NaCl, 50 mM Tris-HCl pH 8.0, 1% NP-40, 0.5% Na-deoxycholate, 0.1% SDS, 10 mM EDTA) containing protease inhibitor cocktail complete mini (Boehringer), 5 mM N-ethylmaleimide (NEM), and 1 mM phenylmethylsulfonylfluoride (PMSF) 24 h post transfection and protein samples were added to equal volumes of sample buffer and separated by SDS PAGE. Virus containing supernatants were centrifuged through 20% sucrose cushions (w/v) for 90 min at 20.000 × *g*. Pelleted VLPs were washed with 1 ml cold PBS and lysed in sample buffer.

### Generation of IDE-knock out HeLa TZM-bl cells by CRISPR/Cas9

HeLa TZM-bl IDE knock out cells were generated by CRISPR/Cas9 technology as described elsewhere [89]. Optimal target sequences for the gRNA design were determined *in silico* by using an online tool (http://crispr.mit.edu/). The vector pSpCas9(BB)-2A-Puro (PX459) V2.0 was a gift from Feng Zhang (Addgene plasmid # 62988). Two gRNAs directed against the target sequences GCTGATGACTTATCCGTGGTGGG (gRNA 1) and TGCTGATGACTTATCCGTGGTGG (gRNA 2) were identified and cloned into the vector pSpCas9(BB)-2A-Puro (PX459) V2.0. HeLa TZM-bl *wt* cells were transfected with the vector containing gRNA 1 or gRNA 2, respectively. 48 h after transfection, cells were selected by treatment with 2 μg/ml puromycin for 24 h. Single cells were obtained by serial dilution. After clonal expansion, the cells were analyzed for enzymatic IDE activity and IDE immunostaining.

### SDS PAGE and western blotting

Protein samples were separated by SDS-PAGE [90] and subsequently transferred onto PVDF or nitrocellulose membranes (GE Healthcare). Membranes were blocked with 3% bovine serum albumin and incubated with the appropriate primary antibody (Ab). Gag was detected by a rabbit Ab recognizing p24 (Seramun). Anti-AU1 (Covance), anti-IDE (Calbiochem) or anti-GFP (Roche) were used to detect the respective proteins or tags. The p6-specific Ab has been described earlier [18]. Antisera for detection of SIV and HIV-2 p6 were generated by immunizing rabbits with the corresponding synthetic peptides (Seramun). As loading control, blots were stained with Abs specific for β-actin (Sigma), ribosomal P-antigen (ribP0, ImmunoVision) or HSP70/90 (Santa Cruz Biotechnology). The *anti-*mouse and *anti-*rabbit secondary Abs coupled to HRP were obtained from Dianova.

### Flow cytometry

For detection of H2-K^b^-bound SL-epitope, cells were stained with the allophycocyanin (APC)-conjugated 25D1.16 mAb (eBioscience) diluted 1:100 in FACS buffer (5% [v/v] FCS, 0.02% [v/v] NaN_3_ in PBS). For detection of total H2-K^b^ molecules, cells were incubated with hybridoma cell culture supernatant containing the mAb B8-24-3 [27], followed by staining with secondary Alexa 647-conjugated anti-mouse Ab (Invitrogen). Flow cytometry was performed on a FACSCalibur using CellQuest software (BD Bioscience). Data were analyzed by using the FACS Express V3 software (De Novo Software).

### *In vitro* degradation assays

For generation of S10, confluent dishes with HeLa cells were extensively washed and subsequently lysed with a modified S10 buffer (250 mM sucrose, 80 mM KCl, 10 mM Tris pH 7.6, 0.1% (v/v) Triton X-100 [91]). Debris and nuclei were removed by centrifugation at 10.000 × g for 10 min. Protein content was determined by BCA-Assay (Pierce) and aliquots stored at -80°C.

Typically 5 μg S10 were incubated with inhibitor or buffer and 20–100 ng *s*p6 at 37°C for 30 min. Reactions were stopped by addition of sample buffer and incubation at 95°C for 5 min. Remaining p6 was detected by Western blotting using an anti-p6 rabbit antiserum [18]. Alternatively, 10 ng of BODIPY FL-labeled *s*p6 (*s*p6BY) were used as substrate and detected directly in the polyacrylamide gel by fluorescence scanning. In some experiments the amount of p6 was densitometrically quantified with the software AIDA (Raytest). For the *in vitro* degradation of amyloid-β 10 ng of Aβ-FITC (abcam) were used analogously to *s*p6BY. For the *in vitro* degradation of viral p6, VLPs were pelleted and lysed in IDE enzyme buffer (150 mM NaCl, 50 mM Tris pH 7.4, 0.5% (v/v) Triton X-100). The p6 content was estimated by Western blotting using a dilution series of *s*p6 with known concentrations. Equivalents of 15 ng were used for digestion experiments and subsequently detected by Western blotting. Experiments carried out with recombinant IDE (Calbiochem) were performed in IDE enzyme buffer. Recombinant FITC-labeled insulin was purchased from Sigma.

### Inhibitors

Bestatin, Chymostatin, E-64, Leupeptin, Pepstatin, Phosphoramidon, Pefabloc SC, EDTA, Aprotinin, Antipain were purchased from Roche as part of a protease inhibitors set and used at the following concentrations: 74 μM Antipain; 130 μM Bestatin; 100 μM Chymostatin; 28 μM E-64; 1 μM Leupeptin; 1 μM Pepstatin; 600 nM Phosphoramidon; 4 mM Pefabloc SC; 1.3 mM EDTA; 0.3 mM Aprotinin. The additional compounds ATP (Boehringer), Bacitracin (Applichem), insulin from bovine pancreas (Sigma), PMSF (Sigma), 6bK [45] (Phoenix Pharmaceuticals and Tocris), TPEN (Calbiochem), and NEM (Roche) were purchased from companies indicated.

### siRNA

IDE-specific siRNA (Life Technologies, sense: GUCUGUUAUCAGAACUUAAtt, antisense UUAAGUUCUGAUAACAGACtt) was transfected in HeLa cells with Lipofectamine RNAiMAX (Life Technologies) according to the manufacturer’s recommendations.

### Peptide synthesis and mass spectrometry

The synthesis of the peptide was performed on a CEM Microwave Peptide Synthesizer Liberty 1 on a 0.1 mM scale with 125 mg H-Gln(Trt) Hmpb-Chematrix-resin (capacity 0.47 mmol/g; PCAS BioMatrix Inc, Canada) or 100 mg Gln-TCP resin (capacity 0.5 mmol/g, Intavis, Germany) using the Fmoc-strategy (*N*-(9-fluorenyl)methoxycarbonyl). Couplings were carried out with N, N, N′, N′-Tetramethyl-O-(1H-benzotriazol-1-yl)uronium hexafluoro-phosphate (HBTU) in N-methylpyrrolidone as coupling agent by a temperature of 50°C. Deprotection of the Fmoc group was performed during the complete synthesis with 5% Piperazine, 0.1 M 1-Hydroxybenzotriazole (HOBt) in *N*, *N*-dimethylformamide. The final deprotection from the resin was performed with 95% TFA in water containing 3% triisopropylsilane. The crude peptide was purified by reverse phase HPLC on a 10 μm Phenomenex Gemini C18 column (21.2 x 250 mm, 110 Å) with a linear gradient of 40% A to 65% B in 50 min (A: 1000 ml water, 2 ml TFA; B: 500 ml acetonitrile, 100 ml water, 1 ml TFA) at a flow rate of 15 ml min^-1^ with spectrophotometric monitoring at λ = 220 nm. The fractions were checked by RP-HPLC (Shimadzu LC10) on a Zorbax 300SB C18 column (4.6 x 250 mm, 5 μ, 300 Å) with a linear gradient of 10 to 100% B over 45 min and mass spectrometry (Voyager DE PRO MALDI-TOF mass spectrometer, linear mode). In the case of chromophoric peptides 1.2 equivalents of Bodipy FLN (Molecular Probes) in DMF would give to 1 equivalent of peptide in PBS-buffer pH 7 (final concentration 2.2 mM). The reaction was monitored by HPLC. After 1 h, the reaction was finished and the peptide was purified again. Peptide sequence for synthetic HIV-2 p6 was derived from the isolate ROD10 and SIV p6 originates from SIVmac239. Synthesis of unlabeled *s*p6 *wt* and *s*p9 has been described earlier [18,29]. The sequence of the p6 mutant E0A has been published previously [24].

### NMR spectroscopy

Prior to NMR analysis of the p6 PTAPPA mutants 2x and 3x, the proteins were dissolved in 600 μl 50% aqueous TFE-D_2_ and transferred to Wilmad 528-PP-7 NMR tubes. TFE-D_2_O was delivered by Cambridge Isotope Laboratories, Inc. (MA, USA). Individual sample concentrations were 650 μM (p6 2x) and 820 μM (p6 3x). The 1D and 2D ^1^H NMR experiments (Total Correlation Spectroscopy (TOCSY), Nuclear Overhauser Effect Spectroscopy (NOESY) and Correlation Spectroscopy (COSY)) were performed at 600.13 MHz on a Bruker Avance 600 MHz instrument equipped with an UltraShield Plus magnet and a triple resonance cryoprobe with gradient unit [54]. The 2D NMR experiments were performed at 300 K without spinning with mixing times of 110 ms for the TOCSY experiments and 250 ms for the NOESY experiments, respectively.

### Viruses

Virus containing cell culture supernatant was harvested 48 h after transfection of 293T cells with proviral constructs and passed through a 0.45 μm pore-size filter. Alternatively, CEMx174 cells were infected with cell culture supernatant containing HIV-1 BK132, HXB2 or IIIB, respectively. After syncytium formation, supernatant was harvested and passed through a 0.45 μm pore-size filter. Virus was pelleted through 20% (w/v) sucrose (20.000 x *g*, 4°C, 90 min) and stocks were normalized for p24, as quantified by p24 ELISA (Aalto). Aliquots were stored at -80°C.

### Infection of cells

For infection, PBMCs were isolated from buffy coats of several blood donors and stimulated with PHA and IL-2 for three days. 1×10^6^ PBMCs were incubated overnight with virus preparations equivalent to 0.031 ng of p24, and cell culture supernatant was collected every second or third day post infection (dpi). Where indicated, the cells were treated with 50 μg/ml insulin or 10 μM 6bK and as part of the medium change on the delineated dpi, fresh insulin was added. Virus replication was assessed by quantification of the virus-associated RT activity by [^32^P]-TTP incorporation, using an oligo(dT)-poly(A) template as described elsewhere [92]. In case of replications with inhibitor treatment, cell viability was determined in parallel by WST-1 assay (Roche) according to the manufacturer’s instructions on the last day of replication. For estimation of the multiplicity of infection (MOI), infectious units of virus stocks were determined by TCID_50_ end point titration in activated PBMCs. The number of HIV-1 target cells was estimated to be 50% of total PBMCs as reported in [93]. The MOI was calculated as the ratio of infectious units per target cells and was ~10^−4^ in case of 0.031 ng p24 of input virus.

### Determination of the replication capacity

To determine the replication capacity of HIV-1 following infection with the indicated HIV-1 variants, the respective replication profiles were depicted as diagram (y-axis: RT activity; x-axis: dpi) and the area under curve was calculated and defined as the corresponding replication capacity of the HIV-1 variants. In every experiment the replication capacity of the untreated *wt* was set to 100% and compared with the indicated variants. Viability of treated cells was assessed on the last day of replication by the water-soluble tetrazolium method. All conditions were not found to diminish cell viability.

## Supporting information

S1 Fig Proteolytic degradation of p6 is conserved within HIV-1. **(A)** 10 ng *s*p6 derived from HIV-1 NL4-3 or *v*p6 isolated from purified virions derived from the HIV-1 isolates AD8, YU-2C, NC7, JC16, BK132, HXB2, and IIIB were incubated with 5 μg S10 extract for 0 or 60 min at 37°C. The reaction was stopped and remaining *s*p6 or *v*p6 was detected by Western blot. Representative Western blots of three independent experiments are shown. **(B)** 10 ng *s*p6BY *wt*, *s*p6BY E0A or *v*p6 S40F, *v*p6 S14N or *v*p6 K27/33R of HIV-1 NL4-3 were incubated with 5 μg S10 extract for the times indicated at 37°C, and remaining *s*p6BY or *v*p6 was detected by fluorescence or Western blot, respectively. Images are representative of three independent experiments. **(C)** Sequence alignment for p6 proteins analyzed by *in vitro* degradation in (A) and (B).
(TIF)

S2 Fig The IDE inhibitors Bacitracin, ATP, and NEM stabilize p6 *in vitro*. 10 ng of *s*p6BY were incubated with 5 μg S10 extract and increasing concentrations of Bacitracin **(A)**, ATP **(B)** or NEM **(C)** for 30 min at 37°C. *s*p6BY was detected by measurement of fluorescence excitation. Values represent the arithmetic mean ± SD of at least three independent experiments.
(TIF)

S3 Fig p6 competes with the *in vitro* degradation of Aβ. **(A)** 10 ng of FITC-labeled amyloid-β 1–40 (Aβ-FITC) were incubated with 5 μg S10 and increasing concentrations of *s*p6 *wt* or insulin for 45 min at 37°C. Aβ-FITC was detected by measurement of fluorescence excitation. The band marked with * represents Aβ-oligomers and has been described earlier [46]. **(B)** Band intensities were quantified for four independent experiments and values represent the arithmetic mean ± SD.
(TIF)

S4 Fig Expression of mature p6 as a transgene in HeLa TZM-bl IDE KO cells. HeLa TZM-bl *wt* or IDE KO cells were transfected with a CMV-driven p6 expression plasmid or empty control vector (pcDNA). Cell lysates were analyzed by Western blotting using a p6-reactive antiserum. Staining for ribP0 served as loading control. Representative Western blots of three independent experiments are shown.
(TIF)

S5 Fig PTAPPA multiplications do not influence the secondary structure of p6. Chemical shift differences (ppm) of the α-protons between the experimental values and those for residues in a random coil for p6 *wt* (**A**) [18] compared with the mutants 2x (**B**) and 3x (**C**) in 50% aqueous TFE-D_2_O at pH 3 at 300 K. Notice that the presence of additional PTAPPA does not influence the positions or the extent of the α-helices of p6. All positive values for N-terminal residues adjacent to proline residues arise from an inherent effect of proline and not out of a structural perturbation [55].
(TIF)

S6 Fig The stability of p6 correlates inversely with the replication capacity of the X4-tropic field isolate HIV-1 BK132. **(A)** A representative replication profile for PHA-IL2-stimulated PBMCs, infected with the X4-tropic field isolate HIV-1 BK132 or mock infected. Replication was assessed by quantification of the virus-associated reverse transcriptase (RT) activity contained in cell culture supernatant collected on the indicated days post infection (dpi). **(B)** The replication capacity of HIV-1 BK132 following infection of PHA-IL2-stimulated PBMCs from 3 different donors and permanent treatment with 10 μM 6bK, 50 μg/ml insulin or the untreated control was assessed by calculating the area under the curve (AUC) from each individual replication profile. The replication capacity of the untreated control in each experiment was set to 100%. Error bars, ± SD.
(TIF)
