# Supplementary figures and images for: Proteolysis of mature HIV-1 p6 Gag protein by the insulin-degrading enzyme (IDE) regulates virus replication in an Env-dependent manner
Source: PLoS One. 2017 Apr 7;12(4):e0174254. doi: 10.1371/journal.pone.0174254 (PMC5384750; doi:10.1371/journal.pone.0174254)

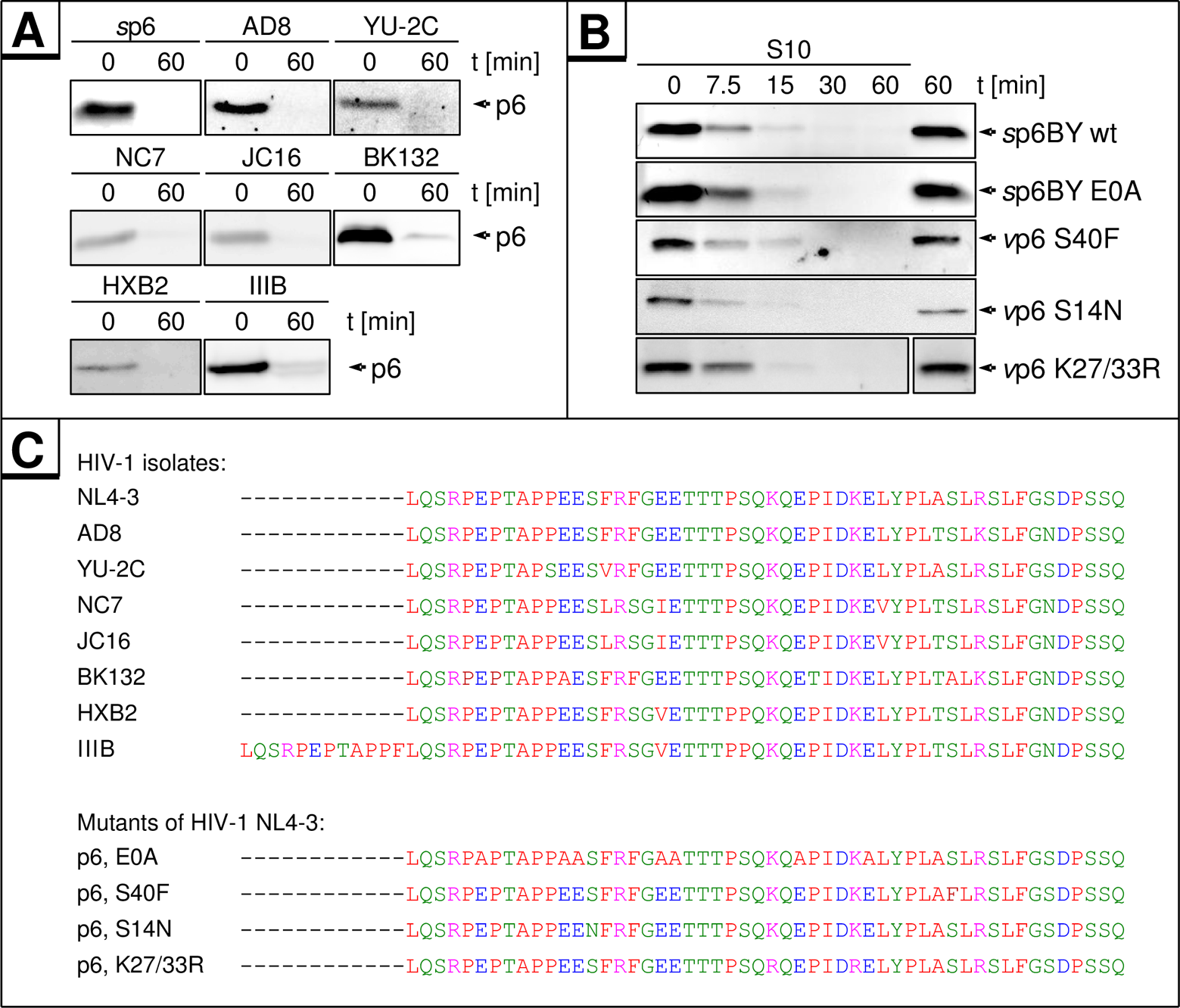

Supplement: S1 Fig — (A) 10 ng sp6 derived from HIV-1 NL4-3 or vp6 isolated from purified virions derived from the HIV-1 isolates AD8, YU-2C, NC7, JC16, BK132, HXB2, and IIIB were incubated with 5 μg S10 extract for 0 or 60 min at 37°C. The reaction was stopped and remaining sp6 or vp6 was detected by Western blot. Representative Western blots of three independent experiments are shown. (B) 10 ng sp6BY wt, sp6BY E0A or vp6 S40F, vp6 S14N or vp6 K27/33R of HIV-1 NL4-3 were incubated with 5 μg S10 extract for the times indicated at 37°C, and remaining sp6BY or vp6 was detected by fluorescence or Western blot, respectively. Images are representative of three independent experiments. (C) Sequence alignment for p6 proteins analyzed by in vitro degradation in (A) and (B). (TIF) [file pone.0174254.s001.tif]

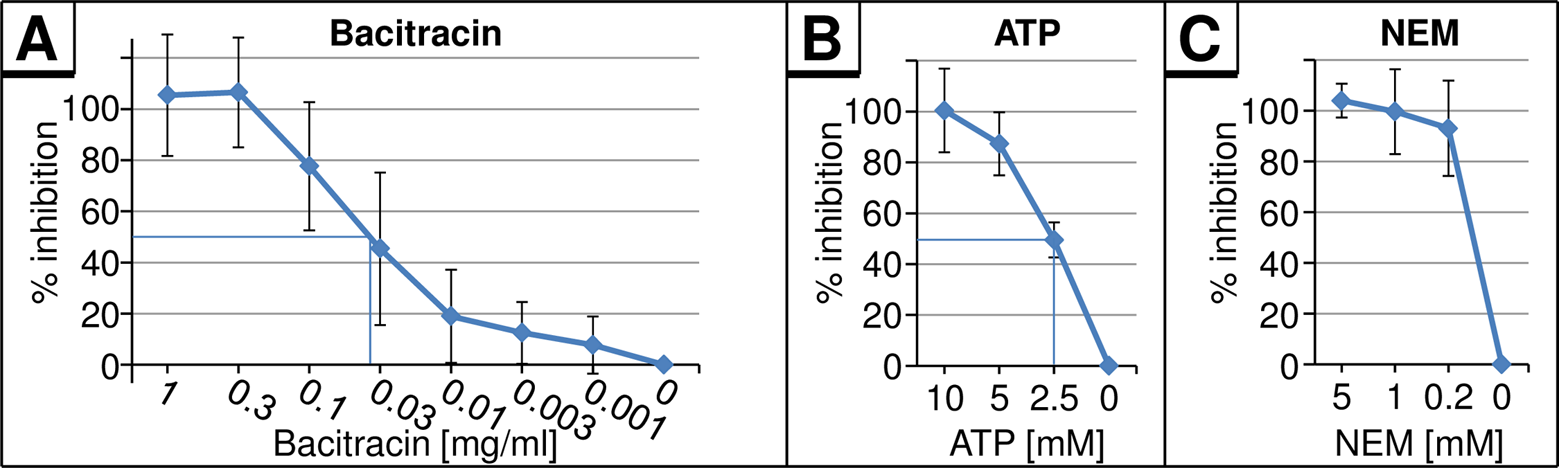

Supplement: S2 Fig — 10 ng of sp6BY were incubated with 5 μg S10 extract and increasing concentrations of Bacitracin (A), ATP (B) or NEM (C) for 30 min at 37°C. sp6BY was detected by measurement of fluorescence excitation. Values represent the arithmetic mean ± SD of at least three independent experiments. (TIF) [file pone.0174254.s002.tif]

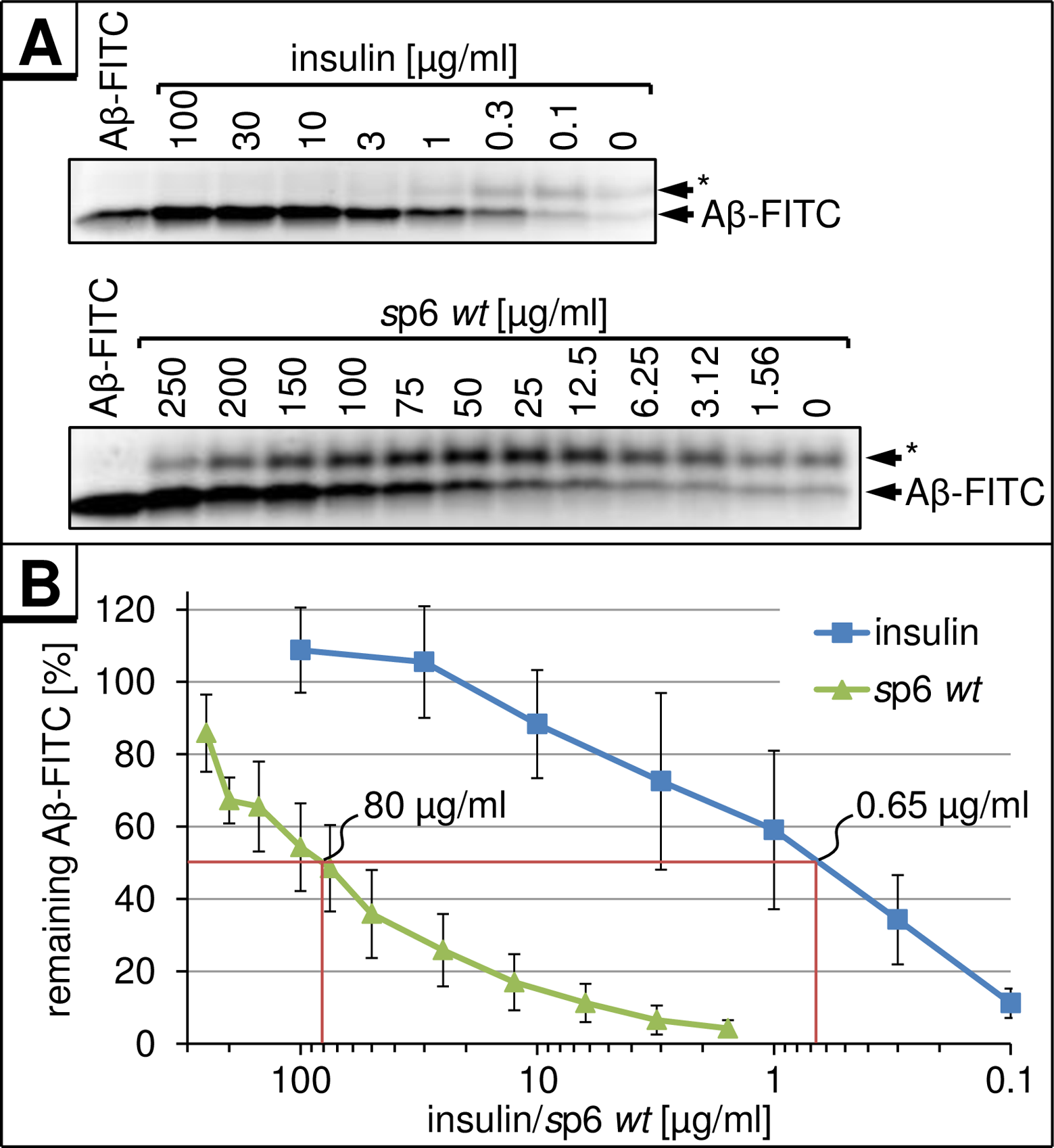

Supplement: S3 Fig — (A) 10 ng of FITC-labeled amyloid-β 1–40 (Aβ-FITC) were incubated with 5 μg S10 and increasing concentrations of sp6 wt or insulin for 45 min at 37°C. Aβ-FITC was detected by measurement of fluorescence excitation. The band marked with * represents Aβ-oligomers and has been described earlier [46]. (B) Band intensities were quantified for four independent experiments and values represent the arithmetic mean ± SD. (TIF) [file pone.0174254.s003.tif]

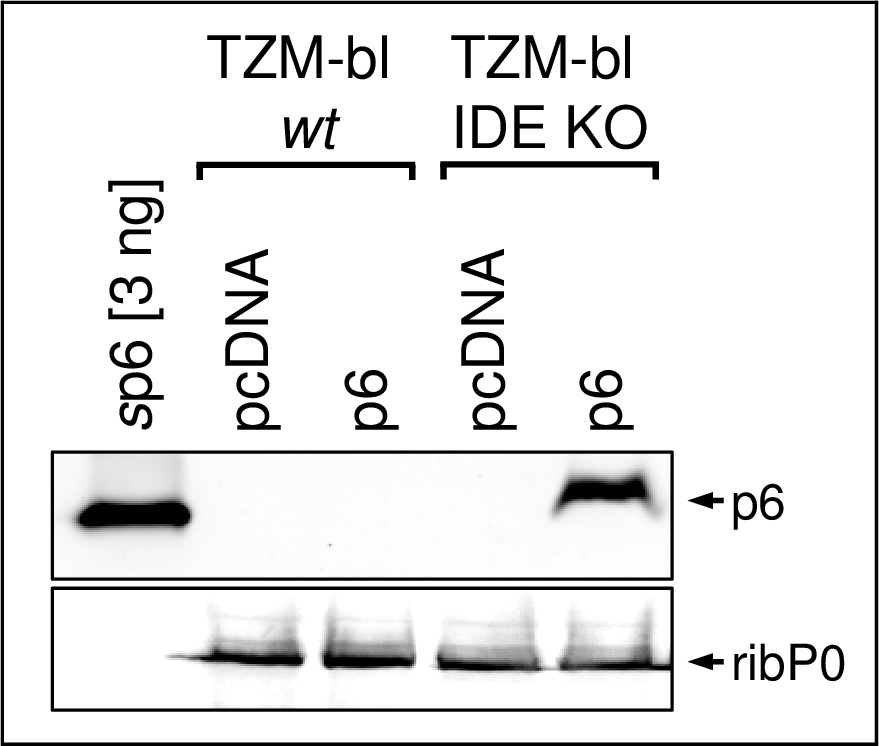

Supplement: S4 Fig — HeLa TZM-bl wt or IDE KO cells were transfected with a CMV-driven p6 expression plasmid or empty control vector (pcDNA). Cell lysates were analyzed by Western blotting using a p6-reactive antiserum. Staining for ribP0 served as loading control. Representative Western blots of three independent experiments are shown. (TIF) [file pone.0174254.s004.tif]

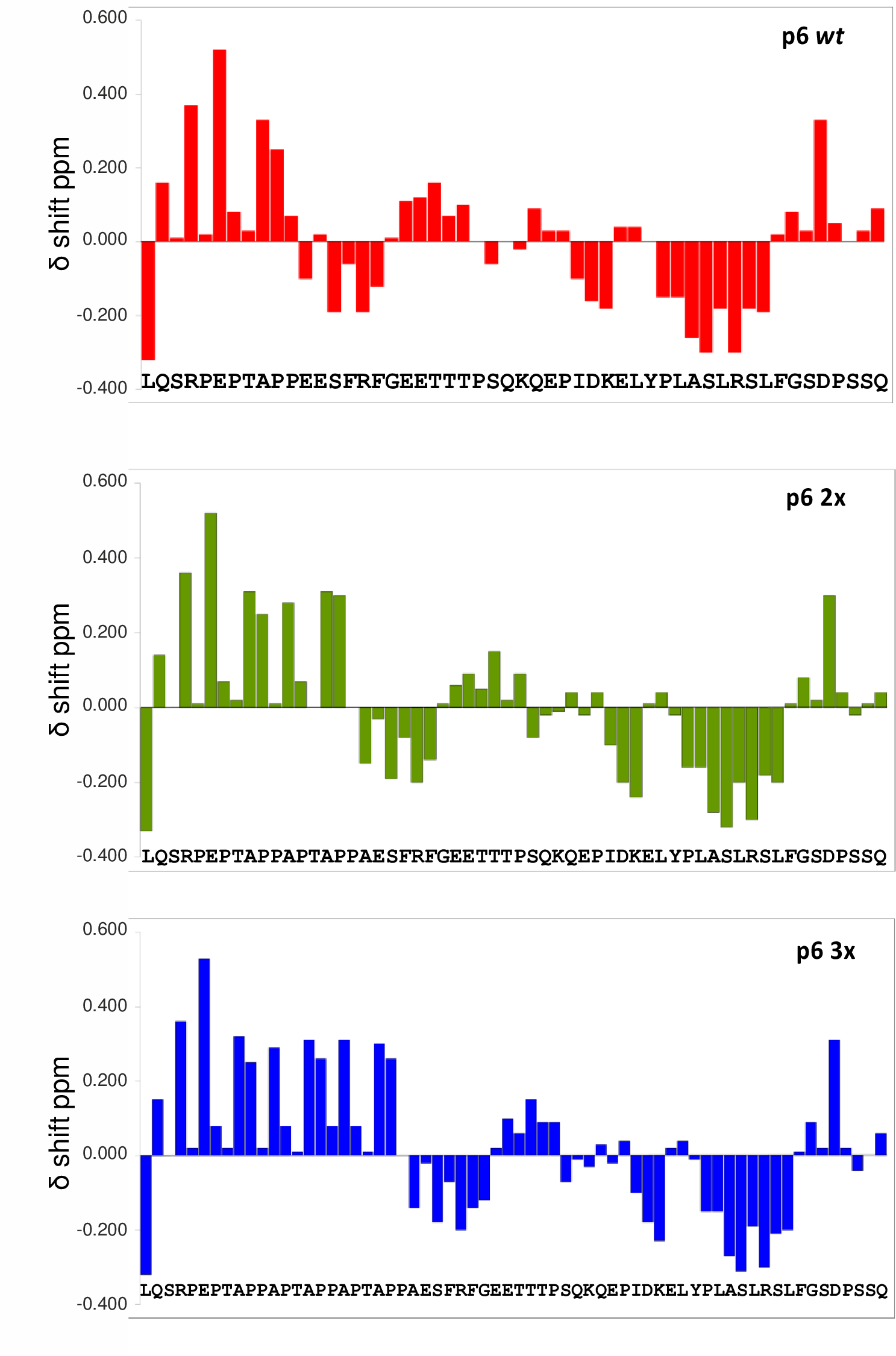

Supplement: S5 Fig — Chemical shift differences (ppm) of the α-protons between the experimental values and those for residues in a random coil for p6 wt (A) [18] compared with the mutants 2x (B) and 3x (C) in 50% aqueous TFE-D2O at pH 3 at 300 K. Notice that the presence of additional PTAPPA does not influence the positions or the extent of the α-helices of p6. All positive values for N-terminal residues adjacent to proline residues arise from an inherent effect of proline and not out of a structural perturbation [55]. (TIF) [file pone.0174254.s005.tif]

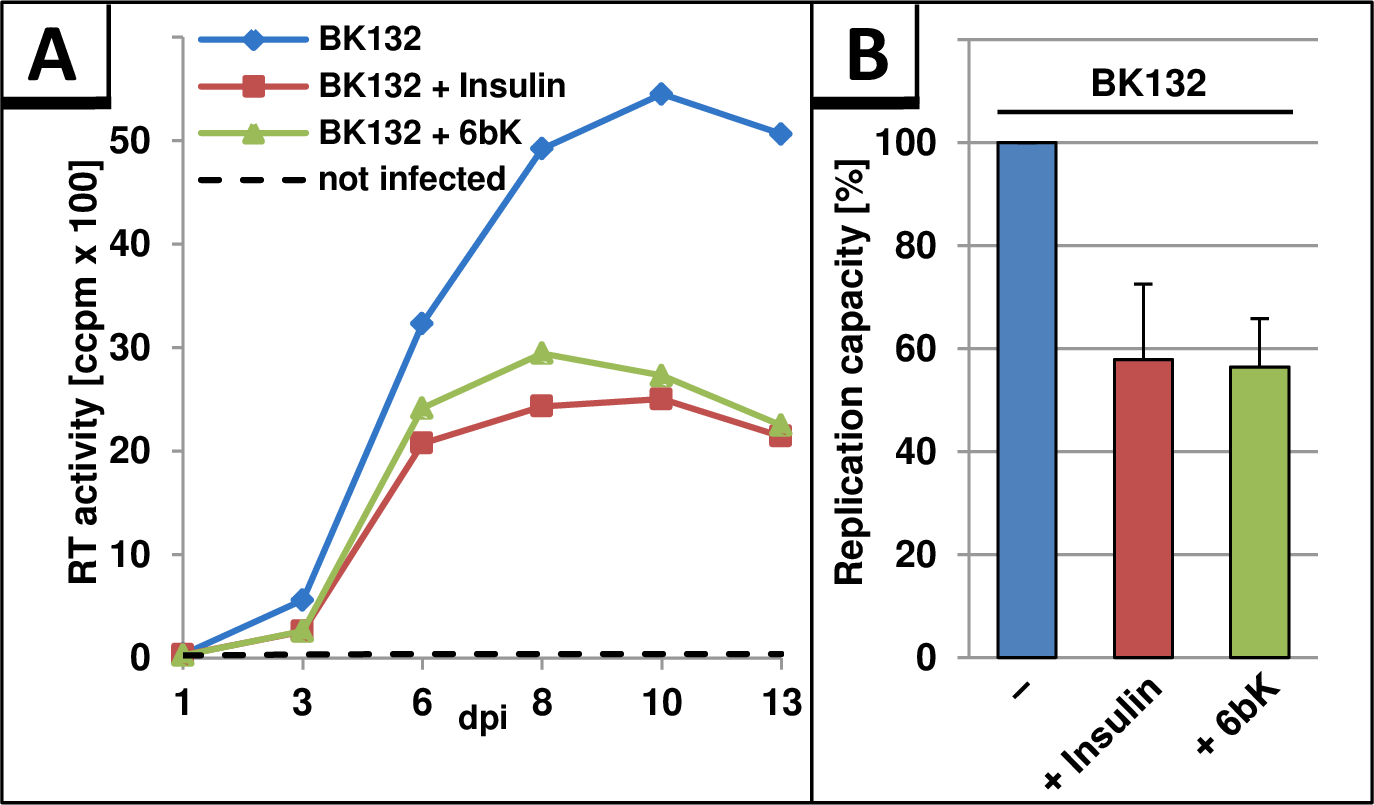

Supplement: S6 Fig — (A) A representative replication profile for PHA-IL2-stimulated PBMCs, infected with the X4-tropic field isolate HIV-1 BK132 or mock infected. Replication was assessed by quantification of the virus-associated reverse transcriptase (RT) activity contained in cell culture supernatant collected on the indicated days post infection (dpi). (B) The replication capacity of HIV-1 BK132 following infection of PHA-IL2-stimulated PBMCs from 3 different donors and permanent treatment with 10 μM 6bK, 50 μg/ml insulin or the untreated control was assessed by calculating the area under the curve (AUC) from each individual replication profile. The replication capacity of the untreated control in each experiment was set to 100%. Error bars, ± SD. (TIF) [file pone.0174254.s006.tif]
